# Favoring the Methane Oxychlorination Reaction over EuOCl by Synergistic Effects with Lanthanum

**DOI:** 10.1021/acscatal.2c00777

**Published:** 2022-04-28

**Authors:** Bas Terlingen, Ramon Oord, Mathieu Ahr, Eline M. Hutter, Coert van Lare, Bert M. Weckhuysen

**Affiliations:** † Inorganic Chemistry and Catalysis Group, Debye Institute for Nanomaterials Science, Utrecht University, Universiteitsweg 99, 3584 CG Utrecht, The Netherlands; ‡ Nobian, Zutphenseweg 10, 7418 AJ Deventer, The Netherlands

**Keywords:** lanthanum, europium, synergy, methane, oxychlorination, reaction mechanism, operando spectroscopy

## Abstract

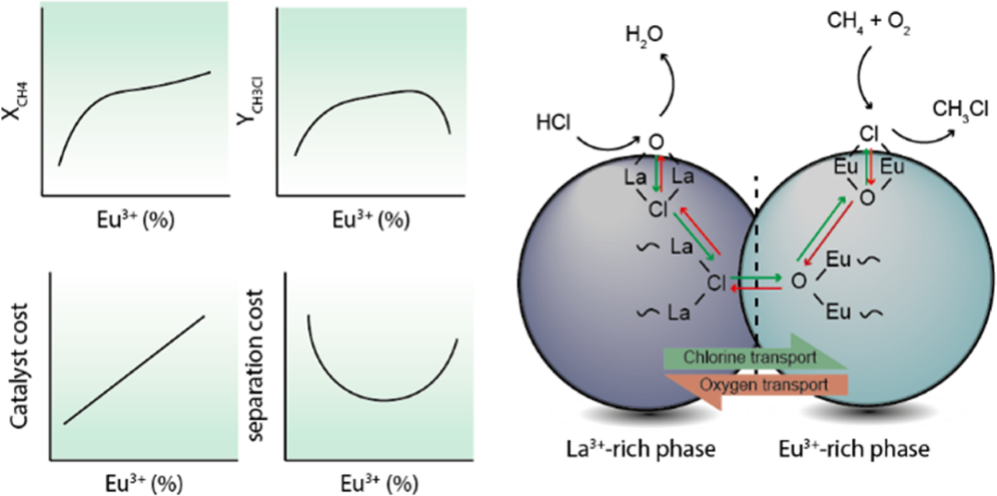

The direct conversion of CH_4_ into fuels and chemicals produces less waste, requires smaller capital investments, and has improved energy efficiency compared to multistep processes. While the methane oxychlorination (MOC) reaction has been given little attention, it offers the potential to achieve high CH_4_ conversion levels at high selectivities. In a continuing effort to design commercially interesting MOC catalysts, we have improved the catalyst design of EuOCl by the partial replacement of Eu^3+^ by La^3+^. A set of catalytic solid solutions of La^3+^ and Eu^3+^ (i.e., La*_x_*Eu_1–*x*_OCl, where *x* = 0, 0.25, 0.50, 0.75, and 1) were synthesized and tested in the MOC reaction. The La^3+^–Eu^3+^ catalysts exhibit an increased CH_3_Cl selectivity (i.e., 54–66 vs 41–52%), a lower CH_2_Cl_2_ selectivity (i.e., 8–24 vs 18–34%), and a comparable CO selectivity (i.e., 11–28 vs 14–28%) compared to EuOCl under the same reaction conditions and varying HCl concentrations in the feed. The La^3+^–Eu^3+^ catalysts possessed a higher CH_4_ conversion rate than when the individual activities of LaOCl and EuOCl are summed with a similar La^3+^/Eu^3+^ ratio (i.e., the linear combination). In the solid solution, La^3+^ is readily chlorinated and acts as a chlorine buffer that can transfer chlorine to the active Eu^3+^ phase, thereby enhancing the activity. The improved catalyst design enhances the CH_3_Cl yield and selectivity and reduces the catalyst cost and the separation cost of the unreacted HCl. These results showcase that, by matching intrinsic material properties, catalyst design can be altered to overcome reaction bottlenecks.

## 1 Introduction

CH_4_ is a relatively cheap and widely available natural resource, but it requires multistep processes to produce fuels and chemicals from it.^1^ Single-step processes conceptually produce less waste, require smaller capital investments, and have improved energy efficiency.^2,3^ However, practical considerations make that none of the direct methane conversion routes have seen industrialization so far.^2^ The key challenges with direct conversion routes that need to be addressed, e.g., low conversion levels and/or poor selectivity, all require better catalyst design.^4,5^ Of the direct conversion routes, methane oxyhalogenation (MOH) reaction has one of the highest potentials to see industrialization due to the moderate reaction temperatures and high conversion levels of CH_4_.^6^ Moreover, a high selectivity toward the desired mono-halogenated methane CH_3_X (where X = Cl, Br, or I) can be achieved.^7,8^ Being able to produce CH_3_X selectively in high quantities is of great interest. The chemical analogy between CH_3_OH and CH_3_X is remarkable^2,9−11^ and makes mono-halogenated methane as valuable as methanol.^5,12,13^ However, relatively little research has been performed on the MOH reaction.^6,12,14^

From the perspective of a circular economy approach, methane oxychlorination (MOC) has the additional advantage of being able to utilize HCl, a byproduct of other chlorination reactions.^15,16^ However, the corrosive and oxidative environment under which the MOC catalysts must operate pose technological challenges and hinder the industrialization of the process.^6,17,18^ A commercially interesting catalyst must be able to operate over prolonged times with high CH_3_Cl selectivity and CH_4_ conversion level.^19^ Furthermore, the selectivity to CO*_x_* needs to be minimized to make optimal use of the chemical feedstock and to lower separation costs.^14^ These aforementioned requirements are challenging, and very little is known about how to fulfill these catalyst requirements.^20,21^ Hence, more work is required to develop suitable MOC catalysts for commercial applications.

A number of catalyst compositions are published in the academic and patent literature, which can be divided into transition metal-based catalysts (e.g., TiO_2_,^8,22^ VPO,^8,22^ FePO_4_,^8^ FeCl_2_/KCl,^23^ ZrO_2_,^24^ and Nb_2_O_5_^22^), noble metal-based catalysts (e.g., RuO_2_,^8,22^ NM/MO^14^ where NM= Ru, Rh, Pd, Ir, Pt, and MO = metal oxide support material), lanthanide-based catalysts (e.g., LaOCl,^25−27^ CeO_2_,^8,12,22,28^ and EuOCl^29^) and bimetallic catalysts (e.g., Cu/K/La,^8,30,31^ FeO*_x_*/CeO_2_,^28,32^ LaVO_4,_^22^ Ce/LaOCl,^17^ Ni/LaOCl,^17^ and Co/LaOCl^17^). None of these groups outperforms any of the other groups by definition, and only a handful of individual solid catalysts were studied in depth. A more fundamental approach to catalyst design needs to be adopted to understand the kinetic and thermodynamic bottlenecks encountered when operating certain catalyst materials.

We recently showed that EuOCl is a promising candidate for the MOC reaction as its performance is stable and, by varying the reaction temperature and feed mixture, also highly tunable.^33^ EuOCl is suitable to be studied under working conditions with *operando* spectroscopy because of the Raman active modes of the material and the photoluminescent properties of Eu^3+^. Hence, we were able to conclude that the chlorination of the catalyst surface was rate limiting. While EuOCl outperformed the other lanthanides tested in our study, a number of improvements need to be made to the catalyst design to have a potential industrial catalyst: (i) improve CH_3_Cl selectivity (S_CH_3_Cl_), preferably at higher CH_4_ conversion levels (X_CH_4__); (ii) reduce catalyst cost by lowering the Eu^3+^ content in the catalyst; and (iii) lower the HCl concentration in the feed while still maintaining a high degree of surface chlorination. A large excess of HCl and unreacted feed are undesired as they result in high separation costs.

In this work, we explore the effect of the partial replacement of Eu^3+^ by La^3+^ on the catalytic performance in the MOC and investigate the apparent synergistic effect between La^3+^ and Eu^3+^. *Operando* Raman spectroscopy previously revealed that the chlorination of EuOCl to EuCl_3_ is a slow process and can be rate limiting during the MOC reaction.^33^ Based on thermodynamic calculations and experimental evidence, LaOCl was selected as a chlorine reservoir for Eu^3+^ as the chlorination from LaOCl to LaCl_3_ occurs readily at low HCl concentrations. La_1–*x*_Eu*_x_*OCl (where *x* = 0, 0.25, 0.50, 0.75, or 1) solid solution catalysts were synthesized and characterized. Incorporation of La^3+^ into EuOCl crystal lattice was favored, since La^3+^ has the same oxidation state and a comparable ionic radius to Eu^3+^. The performance of La_1–*x*_Eu*_x_*OCl materials in the MOC reaction was tested and compared to the benchmark EuOCl. The addition of La^3+^ improved the degree of chlorination of the catalyst, thereby improving the CH_3_Cl yield while preserving the excellent CO selectivity compared to monometallic EuOCl. Furthermore, *operando* luminescence spectroscopy was applied to provide further insight into the chlorination behavior of La^3+^–Eu^3+^ solid solutions. Lastly, physical mixtures of LaOCl and EuOCl were used as catalytic material, showcasing the importance of intimate contact between La^3+^ and Eu^3+^ in the MOC reaction. This resulted in the enhancement of the catalytic performance, approaching the performance of the La^3+^–Eu^3+^ solid solution. Hence, we showcase that, by matching intrinsic material properties, catalyst design can be altered to overcome reaction bottlenecks.

## 2 Experimental Methods

### 2.1 Catalyst Synthesis

The La_1–*x*_Eu*_x_*OCl (where *x* = 0, 0.25, 0.5, 0.75, or 1) catalyst materials under study were prepared by dissolving lanthanum(III) chloride hydrate (LaCl_3_·*x*H_2_O, Alfa Aesar, >99.9%) and/or europium(III) chloride hydrate (EuCl_3_·*x*H_2_O, Alfa Aesar, >99.9%) in ethanol (absolute, VWR), followed by precipitation using stoichiometric amounts of ammonium hydroxide (Fisher Scientific, 25% in H_2_O) at room temperature. After the dropwise addition, the precipitates were stirred for an additional hour and subsequently centrifuged to obtain the gel. Then, the obtained gel was washed with ethanol (absolute, VWR) and dried at 80 °C in air. Lastly, the dried solids were calcined in a static oven at 500 °C for 3 h using a ramp rate of 5 °C/min.

### 2.2 Catalyst Characterization

X-ray diffraction (XRD) patterns were obtained with a Bruker-AXS D8 powder X-ray diffractometer in Bragg–Brentano geometry, using Cu K_α1,2_ = 1.54184 Å, operated at 40 kV. The measurements were carried out between 22 and 65° using a step size of 0.02° and a scan speed of 1 s, with a 2 mm slit for the source. N_2_ adsorption isotherms were measured at −196 °C on a Micromeritics TriStar II Plus instrument. Prior to all measurements, samples were dried at 300 °C in a flow of N_2_. Specific surface areas were calculated using the multipoint Brunauer–Emmett–Teller (BET) method (0.05 < *p*/*p*_0_ < 0.25). Pore volumes were calculated by the *t*-plot method; pore size distributions were obtained by the Barrett–Joyner–Halenda (BJH) analysis; Harkins and Jura thickness model was applied for the *t*-plot and BJH methods.

Inductively coupled plasma-optical emission spectroscopy (ICP-OES) was applied to determine the chemical composition of the catalyst materials, using a SPECTRO CIROS^CCD^ instrument. ICP-OES samples were prepared by destructing the solids in aqua regia.

*Operando* spectroscopy determination of the qualitative EuOCl/EuCl_3_ signal ratio by luminescence spectroscopy was performed with an AvaRaman-532 Hero-Evo instrument (λ = 532 nm, laser output 50 mW, spectral resolution of 10 cm^–1^) equipped with an AvaRaman-PRB-FC-532 probe, capable of withstanding temperatures up to 500 °C. Spectra were collected every minute with the AvaSoft 8 software. The data were subsequently dark corrected. The initial signal was optimized to obtain at least 50% of the saturation value.

### 2.3 Catalyst Testing

All of the catalytic tests and *operando* spectroscopy characterization experiments were performed in a lab-scale continuous-flow fixed-bed reactor quartz reactor. Details on the experimental setup as well as definitions and calculations are reported elsewhere.^33^

Methane oxychlorination reaction: 500 mg of catalyst material (125–425 μm sieve fraction) was loaded in a quartz reactor and heated to 450 °C under N_2_ with a 10 °C/min heating rate. The catalyst was activated in 20% HCl/N_2_ for 2 h prior to catalysis. For the isothermal experiments, the reaction temperature was adjusted to reach X_CH_4__ = 10% for CH_4_/HCl/O_2_/N_2_/He of 2:2:1:1:14. When a stable conversion was reached, the HCl/He ratio was adjusted so that the HCl concentration was increased to 20, 40, 60, and 80 vol % while keeping a constant flow of 20 mL/min. For the ramp experiments, the reactor was brought to 350 °C and the desired feed mixture (i.e., CH_4_/HCl/O_2_/N_2_/He of 2:2:1:1:14 or 2:16:1:1:0 in mL/min) was fed into the reactor. A stabilization period of 30 min was applied, and then the ramp experiment of 1 °C/min was commenced to 550 °C. For the stability tests, the reactor was brought to 450 °C and CH_4_/HCl/O_2_/N_2_/He of 2:2:1:1:14 was fed into the reactor for 4 h. Subsequently, the HCl concentration was increased to 20, 40, 60, and 80 vol % while keeping a constant flow of 20 mL/min. Every HCl concentration was fed for 2 h. To characterize the spent catalysts, the catalysts were dechlorinated at 550 °C for 5 h under CH_4_/HCl/O_2_/N_2_/He of 2:0:4:1:13. The background of this dechlorination step is provided in SI Section S2. For the determination of the apparent activation energy, 250 mg of catalyst (125–425 μm sieve fraction) was loaded in a quartz reactor to 350 °C under N_2_ with a 10 °C/min heating rate. The catalyst was subjected to CH_4_/HCl/O_2_/N_2_/He of 2:2:1:1:14 (in mL/min) for 1 h. The temperature was increased to 550 °C with increments of 10 °C with a heating rate of 5 °C/min and kept at every temperature step for 45 min to obtain the steady-state activity. Only the data points where the methane conversion level was below 10% were considered for fitting the apparent activation energy to avoid heat and mass transfer limitations.

HCl oxidation: 500 mg of catalyst material (125–425 μm sieve fraction) was loaded in a quartz reactor and heated to 450 °C under N_2_ with 10 °C/min. The catalyst was activated in 20% HCl/N_2_ for 2 h prior to catalysis. Temperature-ramp experiments were performed from 350 to 550 °C at a ramp rate of 1 °C/min under the desired feed mixture (i.e., CH_4_/HCl/O_2_/N_2_/He of 0:2:1:1:16 or 0:16:1:1:2 in mL/min).

## 3 Results and Discussion

### 3.1 Catalyst Properties

The synthesized La_1–*x*_Eu*_x_*OCl catalyst material was characterized by N_2_ physisorption, inductively coupled plasma-optical emission spectroscopy (ICP-OES), and X-ray diffraction (XRD) to gain insights into their physicochemical properties (Table 1). The applied base precipitation method yielded catalyst materials with specific surface area (*S*_BET_) and pore volume (*V*_pore_) of the same order of magnitude. The *S*_BET_ ranges between 24.4 and 41.5 m^2^/g, while the *V*_pore_ ranges between 0.06 and 0.23 cm^3^/g. Furthermore, the experimental La^3+^/Eu^3+^ molar ratio obtained from ICP-OES after the precipitation of the bimetallic catalysts is in good agreement with the desired theoretical ratio (Table 1).

**Table 1:** Physicochemical Properties of the As-Synthesized LaOCl, La_0.75_Eu_0.25_OCl, La_0.50_Eu_0.50_OCl, La_0.25_Eu_0.75_OCl, and EuOCl^a^

	physisorption results			phase 1 (La^3+^ rich)			phase 2 (Eu^3+^ rich)		
catalyst material LnOCl where Ln =	*S*_BET_ (m^2^/g)	*V*_pore_ (cm^3^/g)	La^3+^/Eu^3+^ molar ratio (ICP-OES)	position (deg)	La^3+^/Eu^3+^	relative area (%)	position (deg)	La^3+^/Eu^3+^	relative area (%)
La	24.4	0.06		30.62					
La_0.75_Eu_0.25_	39.6	0.22	74:26	30.80	86:14	54	31.02	68.1:31.9 ±1.2	46
La_0.50_Eu_0.50_	41.1	0.18	50:50	30.88	79:21	47	31.42	34.5:65.5 ± 1.3	53
La_0.25_Eu_0.75_	41.5	0.16	24:76	30.99	70:30	21	31.69	16.0:84.0 ±1.7	79
Eu	37.4	0.23					31.91		

a Specific surface area (*S*_BET_) and pore volume (*V*_pore_) were derived based on N_2_ physisorption results. The La^3+^/Eu^3+^ ratios obtained from inductively coupled plasma-optical emission spectroscopy (ICP-OES) corresponded well with the theoretical values. Positions of the deconvoluted (110) X-ray diffraction (XRD) peak, the corresponding La^3+^/Eu^3+^ ratio, and relative area as calculated with Vegard’s Law for as-synthesized La_0.75_Eu_0.25_OCl, La_0.50_Eu_0.50_OCl, and La_0.25_Eu_0.75_OCl are also tabulated.

**Figure 1:**
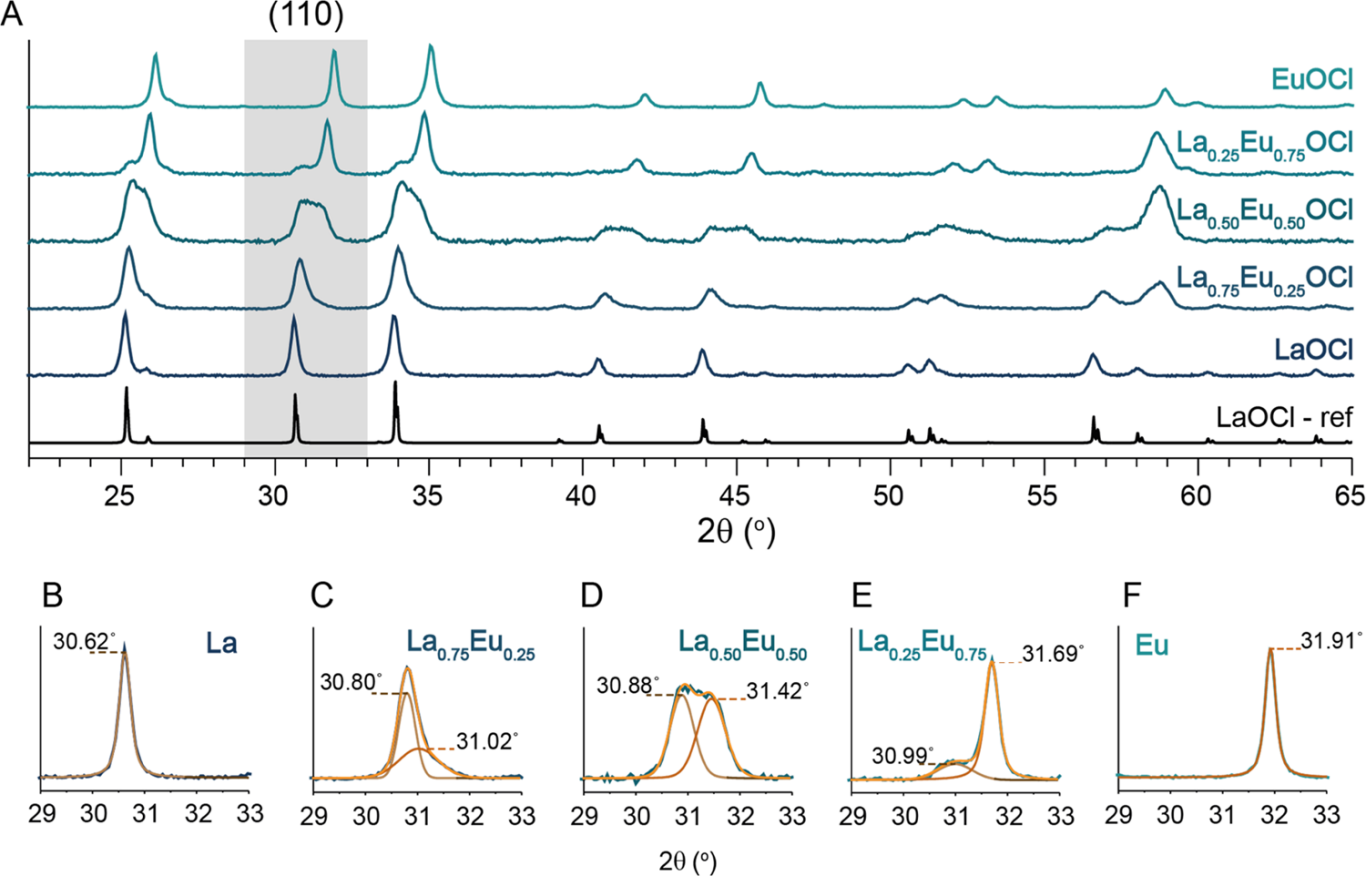
(A) X-ray diffraction (XRD) patterns of the as-synthesized LnOCl catalyst materials under study, including LaOCl, La_0.75_Eu_0.25_OCl, La_0.50_Eu_0.50_OCl, La_0.25_Eu_0.75_OCl, and EuOCl and LaOCl reference pattern (ICDD 00-00800477). (B–F) Zoom-in of the (110) XRD peaks displays the fitted peaks used for determining the degree of La^3+^–Eu^3+^ mixing in Table 1 according to Vegard’s law (see SI Section 1B for the applied procedure).

While ICP-OES provided the elemental ratio of the bulk materials, it did not provide information on the distribution of the two elements throughout the material. XRD was applied to investigate if the desired oxychloride phase was obtained, and if solid solutions of La^3+^ and Eu^3+^ were obtained. The XRD patterns of the as-synthesized catalyst materials are given in Figure 1A. As previously reported, LaOCl and EuOCl are easily synthesized in the oxychloride phase without any noticeable contaminations from other crystalline phases.^33^ Since LaOCl and EuOCl have the same space group, *P*4/*nmm*, and comparable ionic radii,^34^ solid-state ion mixing of the two elements is expected to occur.^35,36^ By deconvolution of the (110) XRD peaks of the as-synthesized LnOCl catalysts (Figure 1B–F) and applying Vegard’s law (see SI Section S1B for more details on the applied procedure), at least two mixed phases were distinguished with varying La^3+^/Eu^3+^ ratios, referred to as phase 1 and phase 2 (Table 1). Noticeable is that for every bimetallic La^3+^–Eu^3+^ catalyst, we obtained one La^3+^-rich phase (*x* > 70%, referred to as phase 1) and one phase with a larger distribution in the La^3+^–Eu^3+^ ratio (phase 2). We hypothesize that LaOCl is precipitated at a higher rate than EuOCl during the synthesis, thereby always obtaining one La^3+^-rich phase. The synthesized catalysts, with known molar ratios and comparable *S*_BET_ and *V*_pore_, enabled us to investigate the role of the La^3+^/Eu^3+^ ratio in the MOC reaction.

### 3.2 Catalytic Performances

**Figure 2:**
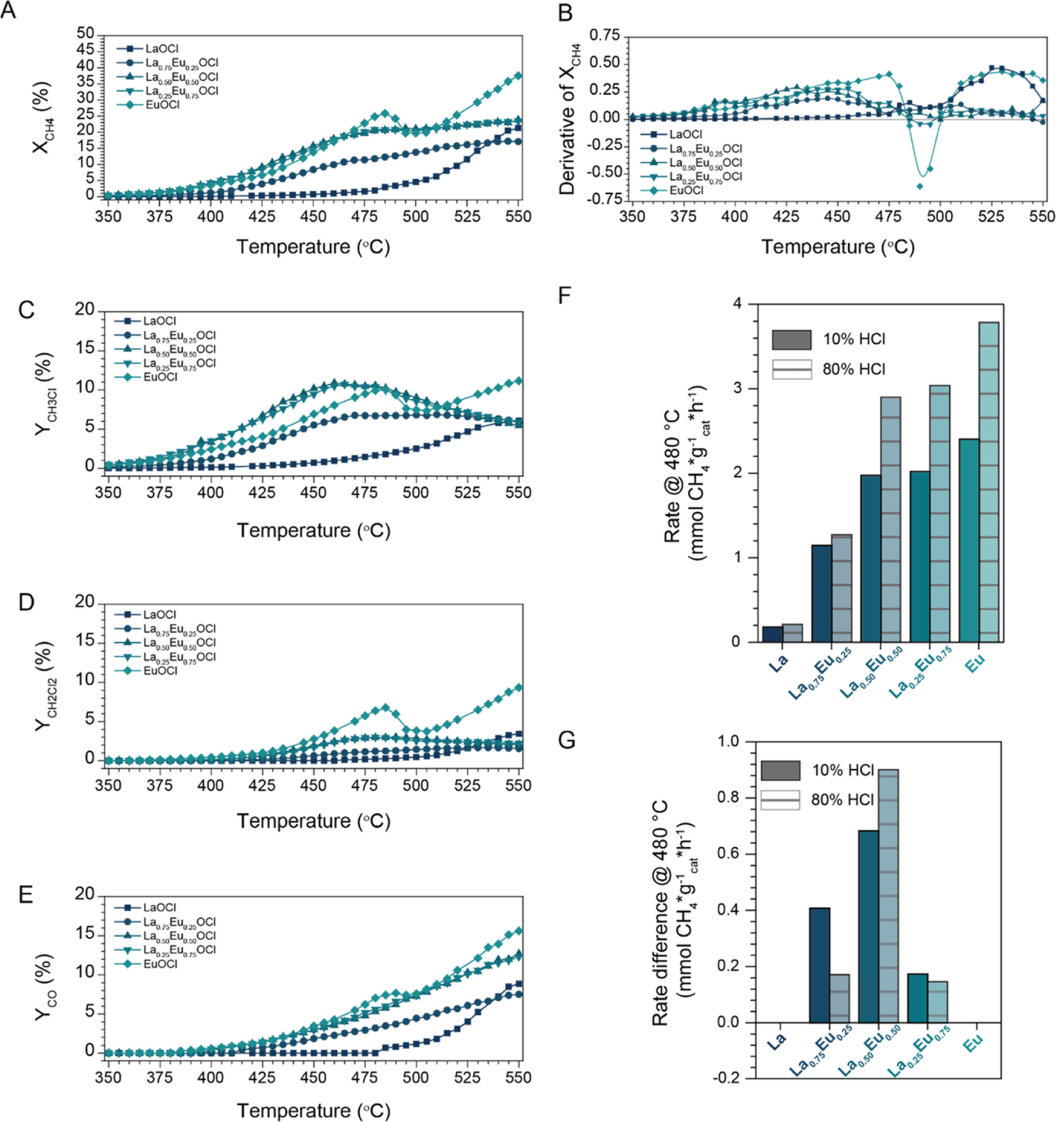
Methane oxychlorination (MOC) experiments for the synthesized La^3+^–Eu^3+^ catalysts. (A) Methane conversion (X_CH_4__) plotted versus the reaction temperature for LaOCl, La_0.75_Eu_0.25_OCl, La_0.50_Eu_0.50_OCl, La_0.25_Eu_0.75_OCl, and EuOCl at 10% HCl. The derivative of the X_CH_4__ versus reaction temperature is plotted in (B). Yields of (C) CH_3_Cl, (D) CH_2_Cl_2_, and (E) CO are plotted versus the reaction temperature at 10% HCl. The CH_4_ conversion rate normalized to the amount of the catalyst is given in (F). Lastly, the rate difference with respect to the linear combination of LaOCl and EuOCl with the same La^3+^/Eu^3+^ ratio is given in (G). Reaction conditions: CH_4_/HCl/O_2_/N_2_/He of 2:2:1:1:14 (10% HCl, in mL/min) or 2:16:1:1:0 (80% HCl, in mL/min), 350–550 °C with a ramp rate of 1 °C/min. The temperature-dependent X_CH_4__ over LaOCl and EuOCl is obtained from ref (33).

Temperature-ramp experiments under MOC reaction conditions were performed to study the catalytic activity trends of the bimetallic La^3+^–Eu^3+^ catalysts. An overview of the catalytic performance of the La^3+^–Eu^3+^ catalysts is given in Figure 2. Individual activity and selectivity versus reaction temperature plots are given in Figure S1. The catalytic performance of pure LaOCl and EuOCl are described elsewhere,^33^ but the plots are given for facile comparison. The reaction temperature at which the catalyst becomes active, referred to as the onset temperature, is determined as the reaction temperature at which the X_CH_4__ > 2%.

The La^3+^–Eu^3+^ catalysts showed many resemblances with respect to each other in terms of catalytic performance as the same qualitative trends could be observed. In general, the bimetallic catalysts showed a steady increase in the X_CH_4__ up to ∼450 °C, after which the X_CH_4__ curve leveled off (Figure 2B). With increasing Eu^3+^ content in the catalyst, the flattening of the X_CH_4__ curve was not only more pronounced but also started at a higher reaction temperature, and thus a higher overall activity was obtained. Also, in terms of the product yield, the same qualitative trends were observed. Y_CH_3_Cl_ reached a maximum at a reaction temperature between 450 and 475 °C, and CH_3_Cl is the dominant product below 500 °C (Figure 2C). Y_CH_2_Cl_2__ was overall quite low, with a maximum yield of ∼3% at 480 °C (Figure 2D). Lastly, Y_CO_ increased steadily over the entire reaction temperature range, reaching its maximum value at 550 °C (Figure 2E). CH_3_Cl and CCl_4_ were detected in minor quantities, with selectivities <3% (Figure S2). No CO_2_ was detected under these reaction conditions.

The bimetallic catalysts showed different catalytic performances compared to their monometallic counterparts. The most striking difference is that the X_CH_4__ of the bimetallic catalysts levels off above 500 °C, while a large increase in X_CH_4__ is observed for both LaOCl and EuOCl (Figure 2A). Furthermore, the observed X_CH_4__ drop for EuOCl, attributed to the dechlorination of EuCl_3_ to EuOCl, is not present when a solid solution is formed between La^3+^ and Eu^3+^ (Figure 2B). Interestingly, the highest Y_CH_3_Cl_ of all catalysts was obtained for La_0.50_Eu_0.50_OCl and La_0.25_Eu_0.75_OCl, reaching a maximum value of 11% at 460 °C. This was significantly higher than the 8% Y_CH_3_Cl_ of EuOCl at the same reaction temperature. This difference was caused by the lower Y_CH_2_Cl_2__ for the La^3+^–Eu^3+^ catalyst compared to EuOCl, as X_CH_4__ and Y_CO_ were similar. One additional advantage of using the bimetallic La^3+^–Eu^3+^ catalysts was that no CO_2_ was detected over the entire tested range, unlike with other catalysts reported in the literature.^8,14,32^

The most balanced performance was observed for La_0.50_Eu_0.50_OCl. The observed X_CH_4__, Y_CH_3_Cl_, Y_CH_2_Cl_2__, and Y_CO_ were similar to La_0.25_Eu_0.75_ and significantly improved compared to La_0.75_Eu_0.25_OCl. This is visualized by normalizing the CH_4_ conversion rate at 480 °C to the amount of catalyst (Figure 2F). A clear trend between the Eu^3+^ content in the catalyst material and the obtained conversion rate is apparent when the activity is normalized to the amount of catalyst and *S*_BET_. The following activity ranking was obtained: EuOCl > La_0.25_Eu_0.75_OCl ∼ La_0.50_Eu_0.50_OCl ≫ La_0.75_Eu_0.25_OCl ≫ LaOCl. Large increments in conversion rates were observed going from LaOCl to La_0.75_Eu_0.25_OCl and to La_0.50_Eu_0.50_OCl, while the CH_4_ conversion rate increments decreased going from La_0.50_Eu_0.50_OCl to EuOCl. Conversely, when the observed activity was corrected for the activity of the linear combination of LaOCl and EuOCl, a synergistic effect between La^3+^ and Eu^3+^ was observed (Figure 2G). The addition of La^3+^ to EuOCl enhanced the activity of Eu^3+^ as all of the La^3+^–Eu^3+^ catalysts possessed a higher conversion rate than when the individual activities of LaOCl and EuOCl are summed with a similar La^3+^/Eu^3+^ ratio (i.e., the linear combination). An optimum was found when an equal amount of La^3+^ and Eu^3+^ was present, as the observed rate difference was the largest. Since monometallic LaOCl showed little activity at this reaction temperature by itself, we hypothesize that LaOCl acts as a chlorine buffer, supplying chlorine to the active Eu^3+^ phase. This effect is caused by the facile chlorination of LaOCl, which increases the degree of chlorination of the catalyst material and hence the activity. The role of La^3+^ and Eu^3+^ is further discussed in Section 3.3. Nevertheless, the observed selectivities for the bimetallic catalysts were not significantly influenced by the catalyst composition (Figure S2). The S_CH_3_Cl_ lied between 53 and 60% for the bimetallic catalysts, which is much better than the S_CH_3_Cl_ of 40% obtained for EuOCl. The S_CO_ in all cases is ∼28% and seems to be governed by the reaction conditions and not by the catalyst composition.

**Figure 3:**
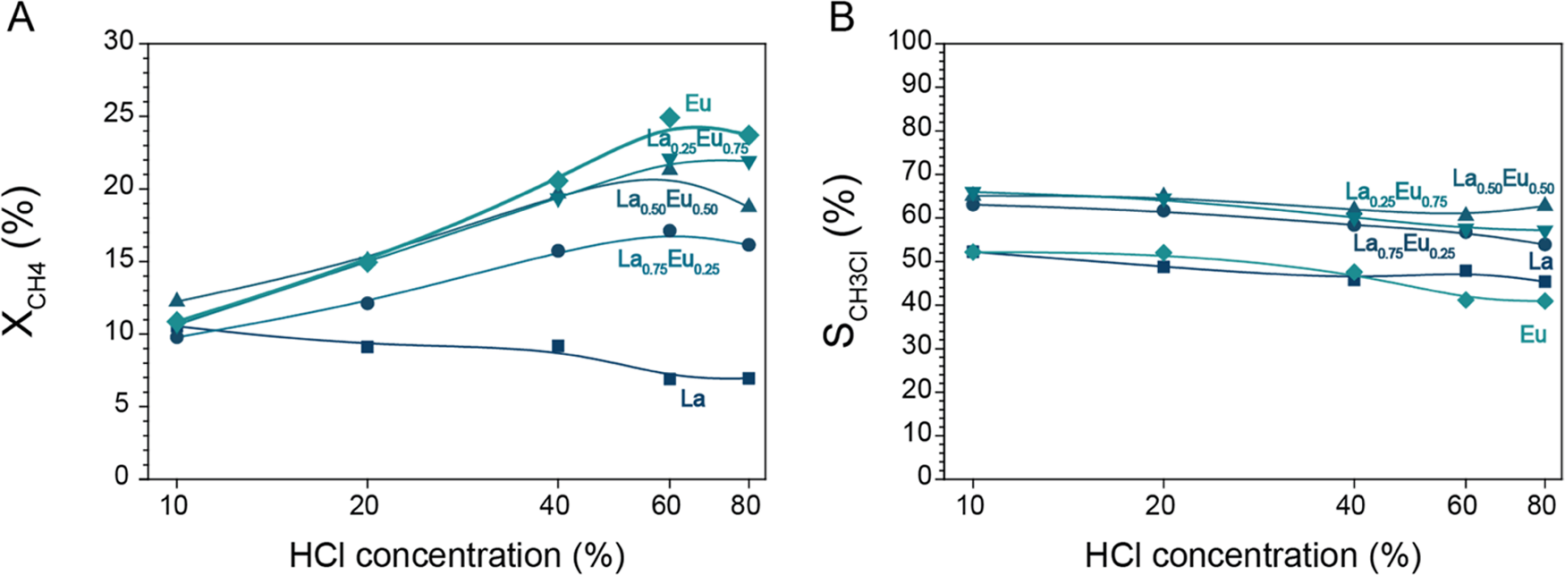
(A) CH_4_ conversion (X_CH_4__) and (B) selectivity toward CH_3_Cl (S_CH_3_Cl_) versus the HCl concentration for LaOCl (*T* = 520 °C), La_0.75_Eu_0.25_OCl (*T* = 475 °C), La_0.50_Eu_0.50_OCl (*T* = 450 °C), La_0.25_Eu_0.75_OCl (*T* = 450 °C), and EuOCl (*T* = 450 °C) in the methane oxychlorination (MOC) reaction. The La^3+^–Eu^3+^ catalyst materials all show increasing X_CH_4__ with increasing HCl concentration. S_CH_3_Cl_ is higher compared to LaOCl and EuOCl over the entire HCl concentration range tested. The temperature was adjusted to reach X_CH_4__ = 10% for CH_4_/HCl/O_2_/N_2_/He of 2:2:1:1:14. When the stable conversion was reached, the HCl/He ratio was adjusted so that the HCl concentration was increased to 20, 40, 60, and 80% while keeping a constant flow of 20 mL/min.

The results presented in Figure 2 show that La^3+^ had a major influence on the activity and selectivity in the MOC reaction. Previously, we applied higher HCl concentrations, i.e., 10–80% HCl in the feed, to boost the catalytic performance of EuOCl.^33^ The catalytic destruction of chloromethanes was circumvented by the high degree of surface chlorination, resulting in improved product selectivity.^9,10,37−41^ With the incorporation of La, similar functionality is incorporated into the catalyst design, and the question arises whether an increment in the HCl concentration is still needed to boost the catalytic performance of La^3+^–Eu^3+^ solid solution catalysts. To investigate the effect of HCl concentration on the La^3+^–Eu^3+^ solid solution catalysts, the reaction temperature was adjusted to obtain X_CH_4__ = 10% after which the HCl concentration in the feed was increased. The X_CH_4__ and S_CH_3_Cl_ are plotted versus the HCl concentration in Figure 3A,B, respectively. The S_CH_2_Cl_2__ and S_CO_ are plotted versus the HCl concentration in Figure S3A,B, respectively. All Eu-containing catalysts were still positively influenced in terms of X_CH_4__ by the increment in HCl concentration. A clear trend in the activity profile was observable going from LaOCl to EuOCl. With increasing Eu^3+^ concentration in the catalyst materials, X_CH_4__ is also proportionally more influenced by the increase in HCl concentration. The reaction selectivity was not influenced drastically by the change in HCl concentration. In general, very small distinctions in terms of selectivity are found comparing the La^3+^–Eu^3+^ catalysts. The La^3+^–Eu^3+^ catalysts follow the same qualitative trend as Eu; only the quantitative performance is more suited for commercial application. Compared to EuOCl, the La^3+^–Eu^3+^ catalysts have an increased S_CH_3_Cl_ (i.e., 54–66 vs 41–52%), lower S_CH_2_Cl_2__ (i.e., 8–24 vs 18–34%), and comparable S_CO_ (i.e., 11–28 vs 14–28%).

To truly compare the catalytic performance of the catalyst material under study, the nonisothermal conversion–selectivity relation was given plotted toward CH_3_Cl and CO (Figure S4). In general, La*_x_*Eu_1–*x*_OCl catalyst materials performed significantly better compared to EuOCl at 10% HCl concentrations as S_CH_3_Cl_ (Figure S4A) and S_CO_ (Figure S4B) were drastically improved at the same conversion level. For example, at X_CH_4__ = 10%, the S_CH_3_Cl_ and S_CO_ of EuOCl were 54 and 25% while for La_0.50_Eu_0.50_OCl, values of 74 and 17% were obtained. Only at high conversion levels (X_CH_4__ > 20%), the EuOCl catalyst performed better than the La*_x_*Eu_1–*x*_OCl catalyst materials, with the important caveat that S_CH_3_Cl_ became too low for practical applications. In the extreme case where the HCl concentration was increased to 80%, the performance of the La*_x_*Eu_1–*x*_OCl catalyst materials was still superior to the performance of EuOCl in terms of S_CH_3_Cl_ (Figure S4C), while the S_CO_ (Figure S4D) were fairly comparable. Here, the La_0.25_Eu_0.75_OCl catalyst performed slightly better than the other La*_x_*Eu_1–*x*_OCl catalyst materials with an S_CH_3_Cl_ and S_CO_ of 74 and 8% at X_CH_4__ = 10%. At the same conversion level, the S_CH_3_Cl_ and S_CO_ of EuOCl were 56 and 6%, respectively. The main difference in product selectivity at 80% HCl concentration is that CH_3_Cl is not further chlorinated to higher chloromethanes for the La*_x_*Eu_1–*x*_OCl catalyst.

The catalytic performance of La_0.50_Eu_0.50_OCl was put in perspective to showcase its excellent performance compared to the catalytic systems reported in literature. For the benchmark catalysts reported in literature, S_CH_3_Cl_ was plotted versus the *T* at which the X_CH_4__ reached 10%, and the reaction rate is also provided (Figure S5). The exact values of the performance of the catalytic systems are tabulated in Table S1. While many catalytic systems show an S_CH_3_Cl_ above 70% at X_CH_4__ = 10% (Figure S5A), a large portion of these catalytic systems are not stable or were not tested for their stability. To comply with the stability criterium, only the catalysts reported as stable in terms of chemical, structural, and catalytic stability are considered (Figure S5B). Now, only a few catalytic systems show S_CH_3_Cl_ above 70% at X_CH_4__ = 10%, making La_0.50_Eu_0.50_OCl a benchmark catalyst. Lastly, the activity was normalized to the volume of the catalyst bed (Figure S5C), evidencing that the La_0.50_Eu_0.50_OCl catalyst is more reactive per unit volume than other catalyst materials reported in the literature.

**Figure 4:**
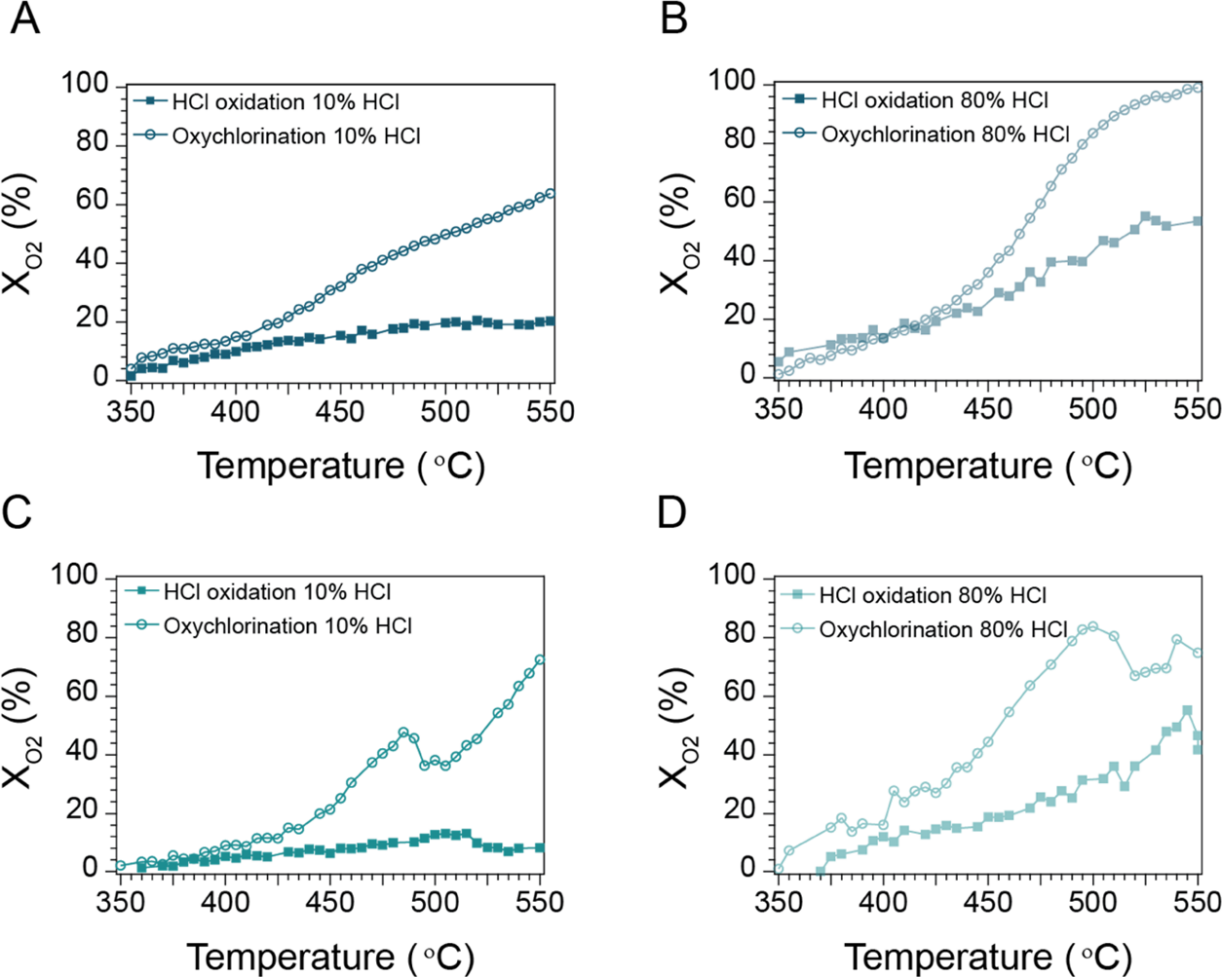
Temperature-ramp experiments where the oxygen conversion (X_O_2__) is plotted versus the reaction temperature for the HCl oxidation reaction (filled squares) and methane oxychlorination (MOC) reaction (open circles) for (A) La_0.5_Eu_0.5_OCl 10% HCl, (B) La_0.5_Eu_0.5_OCl 80% HCl, (C) EuOCl 10% HCl, and (D) EuOCl 80% HCl in the feed. Reaction conditions for the HCl oxidation: CH_4_/HCl/O_2_/N_2_/He of 0:2:1:1:16 (10% HCl, in mL/min) or 0:16:1:1:2 (80% HCl, in mL/min), 350–550 °C with a ramp rate of 1 °C/min. Reaction conditions for the oxychlorination: CH_4_/HCl/O_2_/N_2_/He of 2:2:1:1:14 (10% HCl, in mL/min) or 2:16:1:1:0 (80% HCl, in mL/min), 350–550 °C with a ramp rate of 1 °C/min. The temperature-dependent X_O_2__ over EuOCl was obtained from ref (33).

Lastly, the change in the chemical composition of the catalyst material may alter the reaction mechanism that is responsible for the chlorination of CH_4_. Gas-phase chlorination via tandem reactions, HCl oxidation, and free radical chlorination is in competition with the surface-driven MOC reaction. To investigate the contribution of the gas-phase chlorination to the observed activity, the HCl oxidation performance of La_0.50_Eu_0.50_OCl was tested. The oxygen conversion (X_O_2__) of the HCl oxidation was compared to the X_O_2__ of the MOC reaction under 10 and 80% HCl in the feed in Figure 4A,B, respectively. For facile comparison, the same plots are given for EuOCl obtained from ref (33) in Figure 4C,D, respectively. At 10% HCl, X_O_2__ for La_0.50_Eu_0.50_OCl increased to a reaction temperature of 500 °C, after which it stabilized at the final X_O_2__ value of ∼20%. This was significantly less than the X_O_2__ during the MOC reaction, which gradually increased to a final X_O_2__ value of ∼62%. A discrepancy between the X_O_2__ of the HCl oxidation and MOC was already observed from 405 °C onwards, evidencing that the surface-driven CH_4_ chlorination is the dominant pathway during MOC at 10% HCl. When the HCl concentration was increased to 80% HCl, thereby also increasing the activity of the catalyst material in the MOC, a steeper increase in the X_O_2__ was observed for the HCl oxidation, which gradually increased up to a final X_O_2__ value of ∼53% at 550 °C. The X_O_2__ was significantly higher when the HCl concentration was increased, and the thermal chlorination had a larger contribution to the overall activity. These trends in both HCl oxidation and MOC match well with the trends observed for monometallic EuOCl. The addition of La^3+^ does not influence the HCl oxidation capability of EuOCl qualitatively.

### 3.3 Understanding the Working Mechanism

The catalytic performance of La^3+^–Eu^3+^ solid catalysts showed clear synergetic behavior when compared to either LaOCl or EuOCl. The premise of making La^3+^–Eu^3+^ solid solutions was to improve the chlorination rate of EuOCl, as this chlorination step was found to be rate limiting.^33^ High HCl concentrations in the feed were needed to boost the activity of EuOCl, which is unfavorable in terms of product separation and size of recycle streams. The chlorination and dechlorination behavior of La^3+^ was studied, and we observed that La^3+^ was readily chlorinated to LaCl_3_. Thermodynamic calculations are consistent with this observation, as the chlorination of LnOCl (Ln = lanthanide) to LnCl_3_ is the most facile for LaOCl (Figure S6). Thus, LaOCl most probably functions as a chlorine acceptor/capacitator for the active EuOCl. However, the harsh reaction conditions under which these solid catalysts operate cause many changes in the physicochemical properties over time, and the intimate contact between La^3+^ and Eu^3+^ could be lost. The loss of intimate contact between La^3+^ and Eu^3+^ implies that the exchange of ions between La^3+^ and Eu^3+^ is made more difficult, thereby losing the synergistic effect. Hence, catalyst stability could pose an issue.

**Figure 5:**
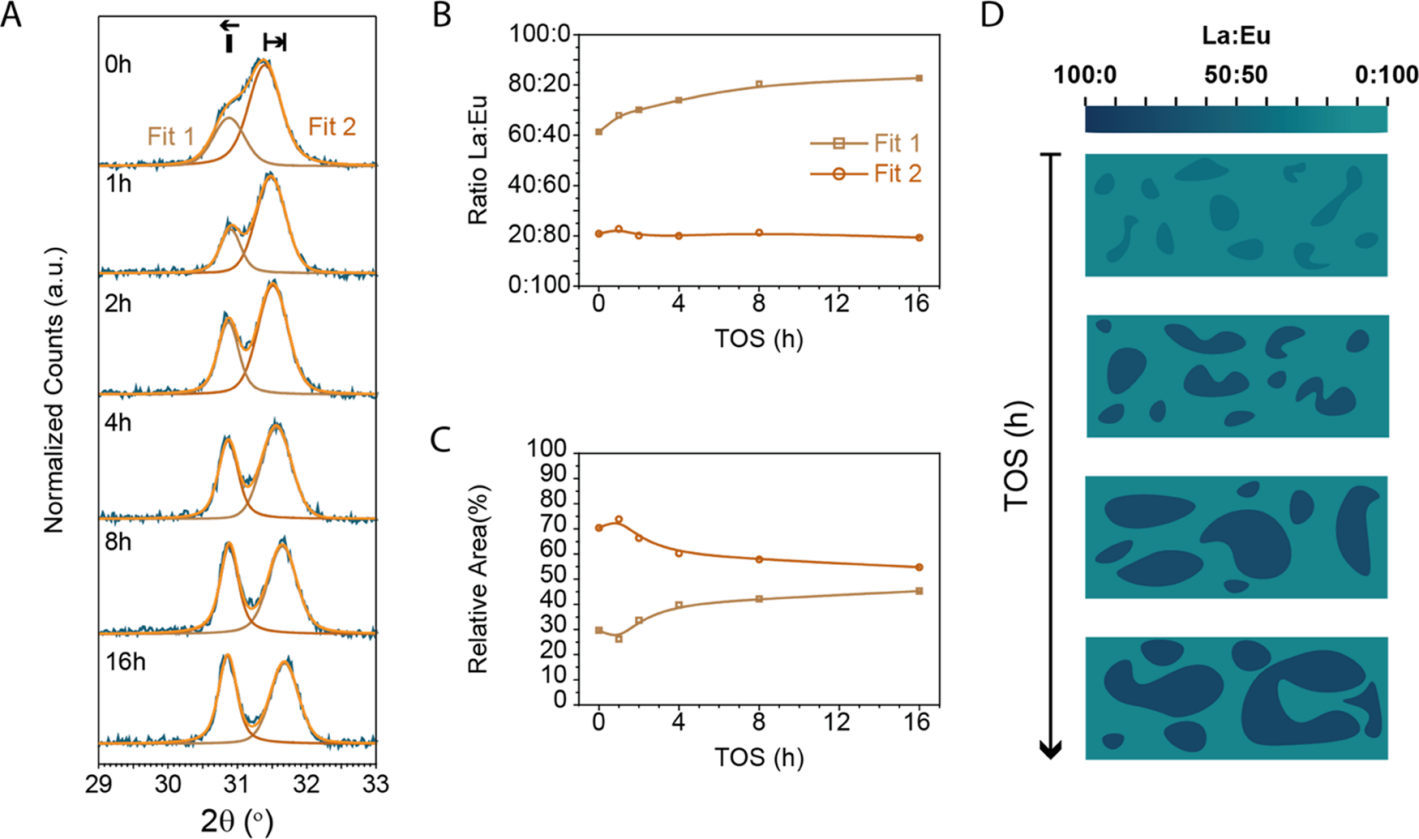
Time series of La_0.50_Eu_0.50_OCl exposed to methane oxychlorination (MOC) conditions to study the phase segregation behavior of La^3+^–Eu^3+^ solid solutions. La_0.50_Eu_0.50_OCl was tested for 1, 2, 4, 8, and 16 h time on stream (TOS) at 450 °C, and the catalyst material was characterized with X-ray diffraction (XRD). The fresh catalyst was loaded into the reactor for every measurement. (A) Zoom-in of the (110) XRD peaks displays phase segregation over time. The obtained (B) La^3+^/Eu^3+^ ratio and (C) relative area of Fit 1 and Fit 2 indicate that the phase segregation predominantly occurs within the first 8 h of reaction. A schematic representation of the phase segregation is depicted in (D), where the La^3+^-rich phase starts to increase in La^3+^ concentration and relative amount.

To analyze whether further phase segregation occurs over time, La_0.50_Eu_0.50_OCl was subjected to MOC conditions for 1, 2, 4, 8, and 16 h, and the postcharacterization results of the chemical composition and structure are presented in Figure 5. Additional transmission electron microscopy (TEM) images of the time series are given in Figure S7 to visualize the morphological changes. Aggregation of particles is visible with increasing time on stream (TOS); however, the dechlorinated catalyst might be altered morphologically, see SI Section S2. The as-synthesized La_0.50_Eu_0.50_OCl displayed two XRD peaks in the region where the (110) lies (Figure 5A), both consisted of La^3+^ and Eu^3+^ (Figure 5B). Over time, the La^3+^-rich phase starts to move to lower angles, indicating the further enrichment of this phase with La^3+^. The Eu^3+^-rich phase, however, does not change in chemical composition (±2% over the entire duration). Simultaneous to the segregation is the change in relative peak area where the La^3+^-rich phase gained in relative peak area. The largest differences were observed in the first 8 h, where the La^3+^/Eu^3+^ ratio of the La^3+^-rich phase changed from 61:39 to 80:20. After 16 h TOS, the La^3+^/Eu^3+^ ratio reached 83:17 for the La^3+^-rich phase.

**Figure 6:**
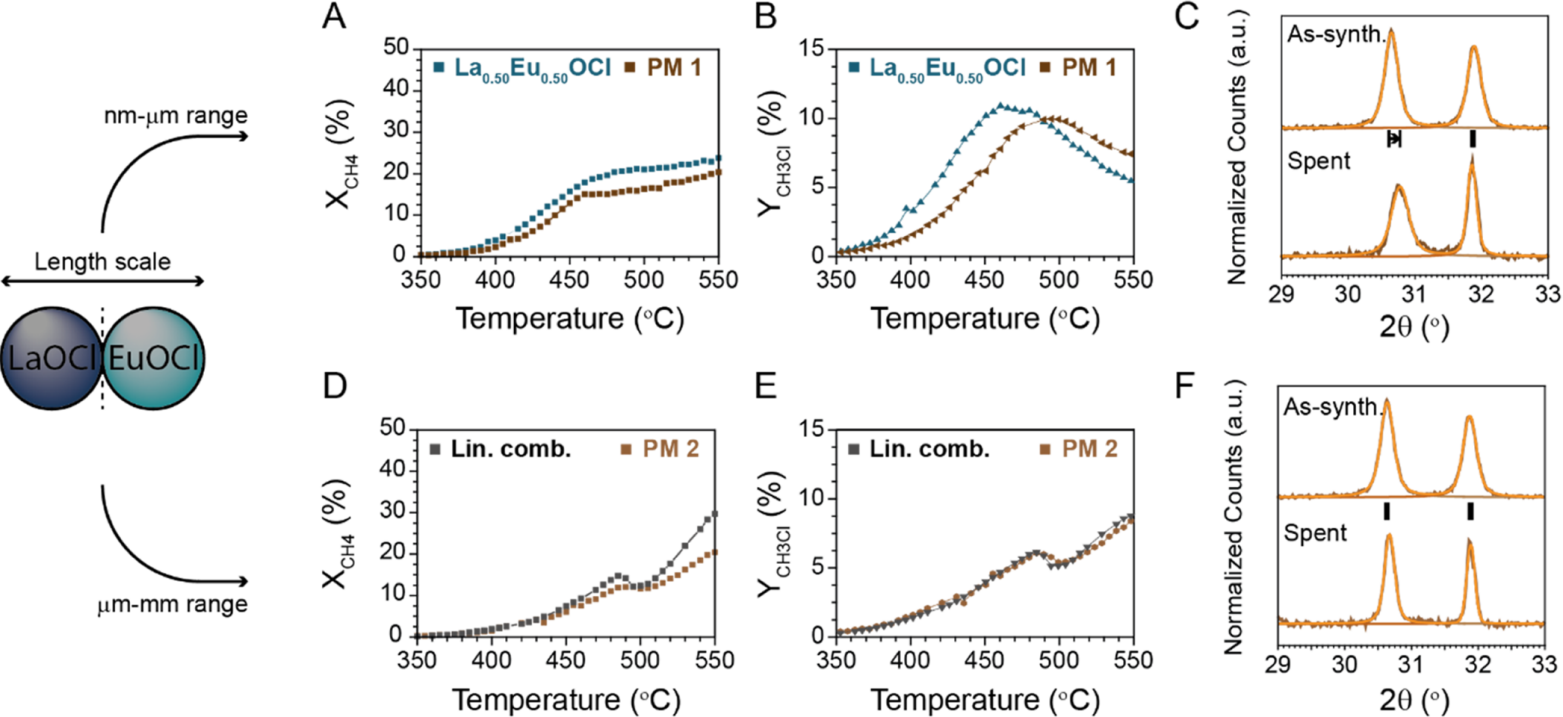
Catalytic performance of PM1 compared to La_0.50_Eu_0.50_OCl and PM2 compared to the linear combination of LaOCl and EuOCl. The (A) X_CH_4__, (B) Y_CH_3_Cl_, and (C) analysis of the (110) X-ray diffraction (XRD) peak of PM1 indicate that the performance of PM1 is very comparable to La_0.50_Eu_0.50_OCl if the intimate contact between LaOCl and EuOCl is established. The (D) X_CH_4__, (E) Y_CH_3_Cl_, and (F) analysis of the (110) XRD peak of PM2 reveal that similar performance to the linear combination of LaOCl and EuOCl is obtained when no intimate contact is established. Reaction conditions: CH_4_/HCl/O_2_/N_2_/He of 2:2:1:1:14 (10% HCl, in mL/min), 350–550 °C with a ramp rate of 1 °C/min.

The observed phase segregation suggests that total phase segregation could occur over prolonged reaction times or harsher reaction conditions, thereby losing the intimate contact between La^3+^ and Eu^3+^. It is unclear if the segregation of these two phases would result in the loss of the synergistic effect between La^3+^ and Eu^3+^. Therefore, to investigate whether this synergistic effect between La^3+^ and Eu^3+^ also exists when the two phases are completely segregated, two physical mixtures of LaOCl and EuOCl were prepared and tested under the same reaction conditions as La_0.50_Eu_0.50_OCl. Physical mixture 1 (PM1) was prepared by sonicating a mixture of LaOCl and EuOCl nanopowders in ethanol, after which the solvent was evaporated and the powder mixture was sieved (125–425 μm size fraction). Intimate mixing of the powders was achieved, but no solid solution was formed. Physical mixture 2 (PM2) was prepared by mixing sieved LaOCl and EuOCl particles (125–425 μm size fraction); hence, no intimate contact is expected. PM1 and PM2 were tested by performing temperature-ramp experiments under 10% HCl and post characterized with XRD. The X_CH_4__, Y_CH_3_Cl_, and the (110) XRD peak of PM1 are presented in Figure 6A–C, respectively, and compared to La_0.50_Eu_0.50_OCl. The same plots as for PM1 were made for PM2 and presented in Figure 6D–F, respectively. A comparison between PM2 and the linear combination of LaOCl and EuOCl is made.

A clear distinction between the observed performance of PM1 and PM2 was apparent. When an intimate contact was achieved, thus in the case of PM1, X_CH_4__ and Y_CH_3_Cl_ much resemble the same trend as observed for La_0.50_Eu_0.50_OCl. Even though some quantitative differences exist, and the overall performance is slightly lower, an enhancement of the activity compared to the linear combination was present (Figure S8). The drop in activity, unique for EuOCl, was not observed, indicating that an intimate contact is established between La^3+^ and Eu^3+^. Surprisingly, mixing of Eu^3+^ in the La^3+^-rich phase occurred, indicated by the shift to higher angles for the La^3+^-rich phase. The La^3+^/Eu^3+^ ratio changed from 100:0 to 88:12. No La^3+^ was incorporated in the EuOCl crystal structure, but migration of Eu^3+^ into LaOCl occurred, possibly because of the higher thermodynamic stability of such phase. The enhancement of activity and mixing of phases did not occur in PM2, when no intimate contact between La^3+^ and Eu^3+^ was present. The activity profile and selectivity of PM2 much resembled a linear combination of the activity of monometallic LaOCl and EuOCl. The drop in activity does occur for this catalyst, which is characteristic of monometallic EuOCl. Furthermore, XRD patterns reveal that no mixing of Eu^3+^ and La^3+^ occurred at these reaction conditions and reaction times. The premise of mixing La^3+^ and Eu^3+^ was to accelerate the chlorination rate of the catalyst material, and hence the activity of Eu, by incorporating a chlorine accepting element in the material. At this point, we observed a synergistic effect between La^3+^ and Eu^3+^ and established the fact that the intimate contact between La^3+^ and Eu^3+^ responsible for this synergistic effect will be preserved. However, it is yet unclear what the mechanism behind this synergistic effect is. Furthermore, during the reaction, a La^3+^-rich oxychloride phase with minor amounts of Eu^3+^ and a (almost) pure EuOCl phase was obtained. To unravel the active phase, we looked at the chlorination behavior of Eu^3+^ in different Eu-containing catalysts.

Structural information, combined with the observed activity in the MOC reaction, provides crucial insight into the working mechanism of these MOC catalyst materials. According to our understanding, the oxychlorination reaction consists of two noncatalytic reactions combined to form a catalytic cycle: the chlorination of lanthanide oxychloride (eq 1) and the dechlorination of lanthanide chloride (eq 2)
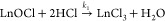
1
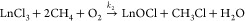
2Many more reactions occur in the complex methane oxychlorination reaction, as, e.g., the dechlorination can also occur via the reaction with H_2_O.^40,41^ For simplicity reasons, the two reaction equations that make up the standard oxychlorination reaction to methyl chloride are given as the main point is the concept of catalyst chlorination and dechlorination. From eqs 1 and 2, it becomes apparent that the state of the catalyst, or the degree of catalyst chlorination, is controlled by . By altering the feed composition, either *k*_1_ or *k*_2_ is directly influenced, which is represented by a change in catalytic performance.

**Figure 7:**
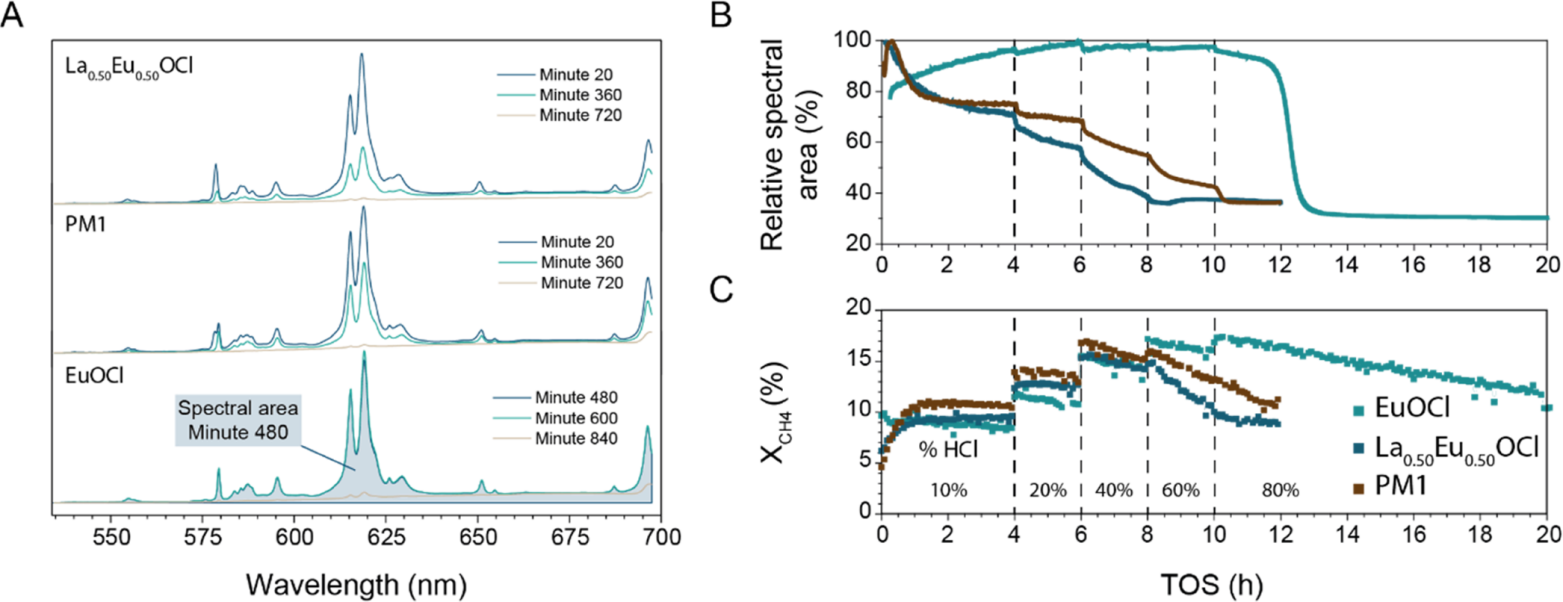
(A) Photoluminescence spectra of La_0.50_Eu_0.50_OCl, PM1, and EuOCl corresponding to the runtimes in (B) show the same behavior to the response in degree of chlorination as observed for EuOCl. The only change appeared in the spectral intensity and not in the shape of the spectrum. The applied integrated spectral area is graphically depicted for EuOCl by the blue area. (B) Relative spectral area of the Eu^3+^ luminescence signal observed during methane oxychlorination (MOC) reaction under varying reaction conditions at 450 °C and (C) corresponding X_CH_4__ plotted versus time on stream (TOS). The incorporation of La^3+^ caused a faster chlorination of Eu^3+^. Reaction conditions: CH_4_/HCl/O_2_/N_2_/He of 2:2:1:1:14 (10% HCl, in mL/min), at 450 °C. Subsequently, the HCl/He ratio was altered to obtain 20, 40, 60, and 80 vol % HCl while keeping a constant flow of 20 mL/min.

The structural information was obtained with *operando* luminescence spectroscopy. The area of the Eu^3+^ luminescence signal was used as a measure for the degree of Eu^3+^ chlorination in previous research.^33^ Since EuCl_3_ shows no luminescence, the decrease in luminescence intensity can be correlated with the degree of chlorination. The Eu^3+^ luminescence spectra of La_0.50_Eu_0.50_OCl and PM1 showed the same emissions as Eu^3+^ in EuOCl and responded in the same manner to a change in degree of chlorination (Figure 7A). Thus, the same analysis can be performed to show the qualitative trends in the degree of chlorination of Eu^3+^ in La^3+^–Eu^3+^ catalyst materials.

When considering EuOCl, very high HCl concentrations and prolonged reaction times were needed to convert EuOCl into EuCl_3_. The relative spectral area of the Eu^3+^ luminescence signal (Figure 7B) and the X_CH_4__ (Figure 7C) are plotted versus the time on stream (TOS), where the HCl concentration in the feed is gradually increased. Here, the first signs of catalyst chlorination started after 10 h and reached their final state after 12 h. The X_CH_4__ gradually increased up to 60% HCl, and a steady downward trend in the X_CH_4__ of EuOCl was visible when the final HCl concentration of 80% was fed, which coincides with previously reported observations that full chlorination deactivates the catalyst material. For EuOCl, only at these very high HCl concentrations, the  > 1, combined with the fact that the activity correlated with the HCl concentration, indicated that the chlorination of the EuOCl surface is the rate-determining step (RDS). Any chlorine present on the surface had reacted before it could diffuse to the bulk; hence, no phase change was observed. If the surface chlorination would not be rate limiting, increasing the HCl concentration would not result in an increase in the activity.

We applied the same principle for La^3+^–Eu^3+^ catalysts to show that La^3+^ addition heavily affects the rate of EuOCl chlorination and thus the rate-determining step. When La^3+^ was in close proximity to Eu^3+^, more facile catalyst chlorination was observed. The highest chlorination rate was observed for La_0.50_Eu_0.50_OCl, as the integrated spectral area already shows a decreasing trend with 10% HCl in MOC reaction conditions. Right from the start,  > 1. This is remarkable, as EuOCl was proven to be difficult to chlorinate under these conditions. The chlorination continued with an increasing rate when the HCl concentration was further increased up to 8 h, where it reached its final state. Complete chlorination was achieved, as no emissions from EuOCl could be detected anymore. Interestingly, up to 8 h, X_CH_4__ increased from 9 to 15%, after which it decreased back to 9% after reaching full chlorination. Qualitatively, the same trend was observed for PM1, but chlorination of the catalyst material occurred at a slower rate. The catalyst material was fully chlorinated after 10 h.

**Scheme 1:**
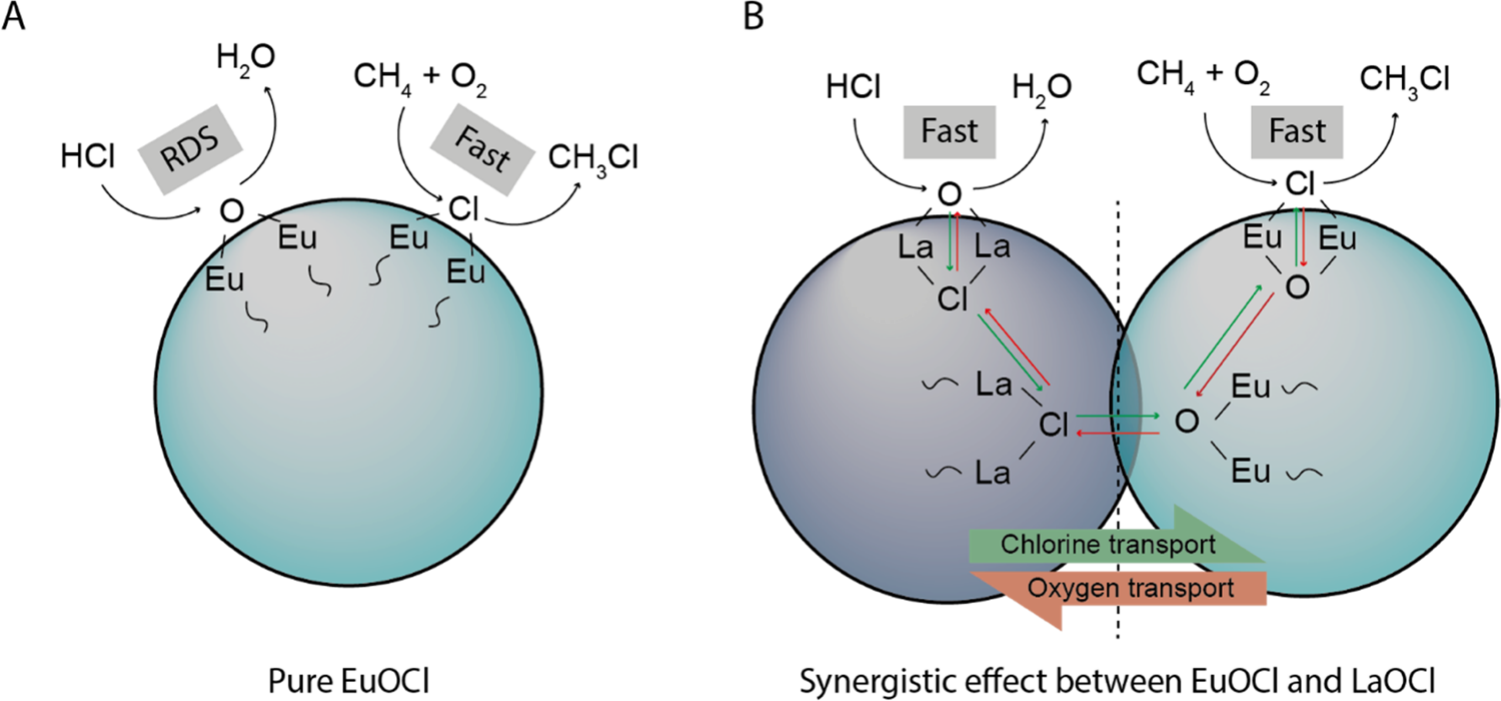
Schematic Representation of the Role of (A) EuOCl and (B) Combination of LaOCl and EuOCl Exhibiting a Synergistic Effect in the Methane Oxychlorination (MOC) Reaction For EuOCl, the rate-determining step (RDS) is the chlorination of the catalyst surface. When La^3+^-rich and Eu^3+^-rich phases are in close proximity to each other, the exchange of ions can occur. The rate-determining step, the chlorination of EuOCl, is accelerated by the presence of LaOCl. The oxygen on the LaOCl surface is replaced with Cl by the reaction with HCl. Subsequently, the excess Cl is transferred to the Cl-deficient EuOCl, after which it is transferred to the surface of the EuOCl phase. The Cl is reacted with CH_4_ and O_2_ on the catalyst surface, leaving an O^2–^ group. Conversely, O^2–^ travels the reverse path.

A crucial observation is that a fast chlorination of Eu^3+^ was expected for La_0.50_Eu_0.50_OCl but not for PM1. PM1 showed no incorporation of La^3+^ into the EuOCl phase (Figure 6), and therefore the same trend as for pure EuOCl would be expected. However, the excellent particle mixing of LaOCl and EuOCl heavily influenced the rate of chlorination of the pure EuOCl. This showcases that the ions in these materials are very mobile, and that facile exchange of ions occurs when the two phases are within close proximity. The apparent activation energy (*E*_app_) of La_0.50_Eu_0.50_OCl (126 kJ/mol) was very comparable to the *E*_app_ of EuOCl (120 kJ/mol), suggesting that the energy needed for the reaction was not altered (Figure S9). A hypothesis on the process of ion exchange is schematically depicted in Scheme 1, responsible for the observed synergistic effect in catalysis. In the case where only EuOCl is present (Scheme 1A), the rate-determining step (RDS) is eq 1. The dechlorination of the catalyst surface is rapid, and therefore the bulk stays in the dechlorinated state. In the case where both Eu^3+^ and La^3+^ are present (Scheme 1B), ion exchange through the bulk occurs. LaOCl, acting as a Cl^–^ acceptor/capacitator, is rapidly chlorinated by the reaction with HCl. Subsequently, the mobile excess Cl^–^ is transferred to the Cl-deficient EuOCl, where an exchange with O^2–^ occurs. The Cl^–^ is reacted with CH_4_ and O_2_ on the EuOCl catalyst surface, replenishing the O^2–^ group. While LaOCl and EuOCl individually are active in the MOC, both capable of surface chlorination and CH_4_ activation, the process of ion exchange is accelerated. Hence, PM1 also exhibited synergistic effects when tested for its MOC performance.

**Figure 8:**
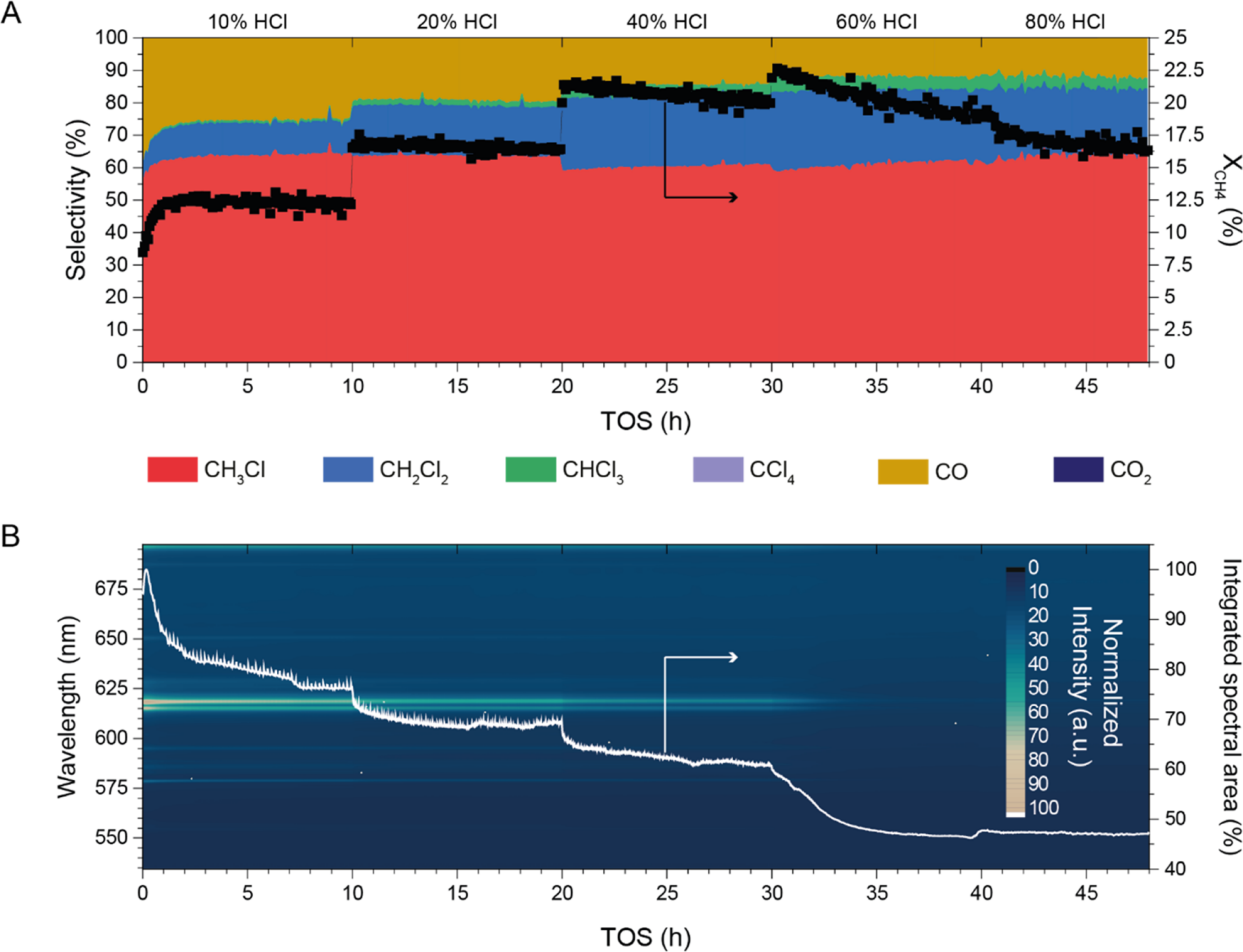
Stability test of La_0.50_Eu_0.50_OCl at 450 °C while varying the HCl concentration in the feed every 10 h. (A) X_CH_4__ and S_CH_3_Cl_, S_CH_2_Cl_2__, S_CHCl_3__, S_CCl_4__, S_CO_, and S_CO_2__ are plotted versus time on stream (TOS). (B) Operando luminescence spectroscopy of Eu^3+^ where the spectra are plotted as a heat map versus the time on stream. Furthermore, the integrated spectral area is plotted versus the time on stream as a measure for the degree of catalyst chlorination. With increasing HCl concentration up to 60%, the X_CH_4__ increased while the S_CO_ and S_CH_3_Cl_ decreased. When 60% HCl was fed in the reactor, the X_CH_4__ sloped down, while simultaneously the catalyst fully chlorinated. Reaction conditions: CH_4_/HCl/O_2_/N_2_/He of 2:2:1:1:14 (10% HCl, in mL/min), at 450 °C. Subsequently, the HCl/He ratio was altered to obtain 20, 40, 60, and 80 vol % HCl while keeping a constant flow of 20 mL/min.

Lastly, the stability of La_0.50_Eu_0.50_OCl under MOC conditions was tested for 48 h at 450 °C under varying HCl concentrations in the feed. Every 10 h, the HCl concentration was increased to find the upper limit under which the catalyst material still exhibits stable performance. Simultaneously, the photoluminescent properties of Eu^3+^ were again used to monitor the degree of EuOCl chlorination. The activity/selectivity in the MOC reaction and the corresponding spectral data are plotted versus the time on stream (TOS) in Figure 8A,B, respectively. La_0.50_Eu_0.50_OCl exhibited very stable X_CH_4__ under 10 and 20% HCl in the MOC reaction, with values of 12 and 16%, respectively. At 40% HCl, a slight downward trend in X_CH_4__ was observable, going from 21 to 19%. The decline was accelerated when the HCl concentration was further increased to 60%. A final X_CH_4__ of 16% was achieved after 48 h. The selectivity in the MOC reaction showed the same stability as observed for X_CH_4__. At 10 and 20% HCl in the feed, an S_CH_3_Cl_ of ∼64% was achieved. When X_CH_4__ showed a decreasing trend, from 60% HCl onwards till the end of the experiment, S_CH_3_Cl_ slightly increased from 59 to 64% in favor of S_CH_2_Cl_2__ and S_CHCl_3__. S_CO_ remained unaltered under these reaction conditions at ∼13%. In line with the trends observable for X_CH_4__ were the observed changes in the spectral intensity. After an initial stabilization period of ∼8 h in which the catalyst is slowly chlorinated, a steady-state composition of the catalyst was achieved as the spectral area did not change until the HCl concentration was further increased to 20%. Again, a stabilization period was observed, which now took roughly 3 h whereafter a steady state was achieved. At 40%, where the X_CH_4__ slowly decreased over time, the integrated spectral area also showed a slightly decreasing slope. From 60% HCl onwards, the catalyst was gradually chlorinated almost to completion (Figure S10). These results suggest that La_0.50_Eu_0.50_OCl is stable in the MOC reaction under the condition that EuOCl is not fully chlorinated to EuCl_3_. This was further evidenced by performing a 100 h during stability test under the same conditions (Figure S11). No sign of deactivation was observed for La_0.50_Eu_0.50_OCl under 10% HCl at 450 °C. Furthermore, the catalytic benefits arising from the synergistic effect between La^3+^ and Eu^3+^, i.e., increased S_CH_3_Cl_, lower S_CH_2_Cl_2__, and similar S_CO_ and X_CH_4__ were preserved.

## 4 Conclusions

In this work, a set of La*_x_*Eu_1–*x*_OCl (where *x* = 0, 0.25, 0.50, 0.75, and 1) solid solutions with comparable physicochemical properties were synthesized. An intimate contact between La^3+^ and Eu^3+^ was achieved, as La^3+^ and Eu^3+^ were incorporated into the same crystal structure. However, methane oxychlorination (MOC) conditions caused phase segregation into two phases: a La^3+^-rich phase and a Eu^3+^-rich phase. These phases were still in close contact with one another, exhibiting synergistic effects in the MOC reaction. LaOCl, which readily chlorinates, acts as a chlorine buffer in the EuOCl catalyst and accelerates the catalyst chlorination rate. Transport of chlorides from the La^3+^-rich phase to the active EuOCl is suspected to take place, facilitating the difficult EuOCl chlorination step. This synergistic effect resulted in the fact that all La^3+^–Eu^3+^ solid solution catalysts possessed enhanced activity as compared to the linear combination of LaOCl and EuOCl. Even in absolute terms, the activity of, e.g., La_0.50_Eu_0.50_OCl approached the activity of EuOCl, even though the material contains 50% less of the active Eu^3+^. Furthermore, mixing La^3+^ and Eu^3+^ also significantly improved the observed selectivity. Compared to EuOCl, the La^3+^–Eu^3+^ catalysts have an increased S_CH_3_Cl_ (i.e., 54–66 vs 41–52%), lower S_CH_2_Cl_2__ (i.e., 8–24 vs 18–34%), and comparable S_CO_ (i.e., 11–28 vs 14–28%) under the same reaction conditions and varying HCl concentrations in the feed. Finally, the synergistic effect between La^3+^ and Eu^3+^ can be assured over extended reaction times as the same synergistic effect can be reached by physically mixing LaOCl and EuOCl. This physical mixture showed qualitatively the same trends as La_0.50_Eu_0.50_OCl, and after reaction, incorporation of Eu^3+^ in the LaOCl crystal structure was found. The improved catalyst design by the partial replacement of Eu^3+^ by La^3+^ makes Eu-based catalysts even more attractive for commercial applications as better CH_3_Cl yield and selectivity could be achieved while also reducing the raw material cost of the MOC catalyst.

## Supporting Information Available

Experimental definitions and calculations, Vegard’s law, postcharacterization of the catalyst materials, additional catalytic data, performance and stability overview of the catalyst reported in the academic literature, transmission electron microscopy images, and photoluminescence spectra of La_0.50_Eu_0.50_OCl (PDF)
