# Supplementary material for: Favoring the Methane Oxychlorination Reaction over EuOCl by Synergistic Effects with Lanthanum
Source: ACS Catal. 2022 Apr 28;12(9):5698–710. doi: 10.1021/acscatal.2c00777 (PMC9087184; doi:10.1021/acscatal.2c00777)
Supplement: Supplementary file 1 — cs2c00777_si_001.pdf [file cs2c00777_si_001.pdf]

Favoring the Methane Oxychlorination Reaction over EuOCl by Synergistic Effects with Lanthanum

Bas Terlingen[#], Ramon Oord[#], Mathieu Ahr[§], Eline M. Hutter[#], Coert van Lare[§], Bert M. Weckhuysen[*,#]

[#] Inorganic Chemistry and Catalysis group, Debye institute for Nanomaterials Science, Utrecht University, Universiteitsweg 99, 3584 CG Utrecht, The Netherlands; Email: B.M.Weckhuysen@uu.nl.

[§] Nobian , Zutphenseweg 10, 7418 AJ Deventer, The Netherlands

1. Experimental Definitions and Calculations

a. Conversion, Yield, Selectivity and Carbon Balance

The CH_4 conversion, X_{CH4}, and O_2 conversion, X_{O2}, are calculated according to Eq. S1,

$$X_{\mathrm{a}}(\%) = \frac{x_{\mathrm{a},\,inlet} - (x_{\mathrm{a},\,outlet} * ISCF)}{x_{\mathrm{a},\,inlet}} * 100\%\ (Eq.\,\mathrm{S1})$$

where $x_{\mathrm{a},\,inlet}$, $x_{\mathrm{a},\,outlet}$ and ISCF stand for the volumetric concentration of compound a at the inlet and outlet of the reactor and the Internal Standard Correction Factor, respectively. The yield of product i, Y_i, is calculated according to Eq. S2,

$$Y_i(\%) = \frac{x_i}{x_{CH4,\,inlet}} * 100\% * ISCF\ (Eq.\,\mathrm{S2})$$

where x_i and $ISCF$ stand for the volumetric concentration of carbon containing product I and the Internal Standard Correction Factor, respectively. The selectivity of product i, S_i, is calculated according to Eq. S3.

$$S_i(\%) = \frac{Y_i}{X_{CH4}} * 100\%\ (Eq.\,\mathrm{S3})$$

The CH_4 reaction rate is calculated according to Eq. S4,

$$R_{CH4}\left(\frac{mmol}{h * g_{cat}}\right) = \frac{P * F_T * x_{CH4,inlet} * \frac{X_{CH4}}{100}}{R * T * W_{cat}} (Eq.\,\mathrm{S4})$$

where P, F_T, R, T and W_{cat} stand for the pressure, total flow, gas constant, ambient temperature and catalyst weight. Finally, the carbon balance was calculated and measurements with their carbon balance > ± 5% were removed. The carbon balance was calculated according to Eq. S5.

$$Carbon\ balance = \sum Y(i) + 100 - X_{CH4}\ (Eq.\,\mathrm{S5})$$

b. Determining the Elemental ratio with Vegard's Law

The Origin 2017 multi peak fit tool was used to fit Voigt peaks functions, which in turn were used to determine the (110) X-ray diffraction (XRD) peak positions. This was done for the monometallic catalysts (references) as well as for the bimetallic catalysts and the results are given in Table 1. From the peak position, the interplanar distance d (nm) was calculated according to braggs law, see Eq. S6.

$$\lambda = 2d * \sin(\theta)(Eq.\,S6)$$

Where λ and θ are the wavelenght of the X-ray source (nm) and the angle of the incident light (°) to the plane respectively. With the use of the interplanar distance and the Miller indices, the lattice parameters were then calculated. For the tetragonal LnOCl crystal system, Eq. S7 must be used to determine lattice parameters a and c (nm).

$$\frac{1}{d^2} = \frac{(h^2 + k^2)}{a^2} + \frac{l^2}{c^2}\ (Eq.\,S7)$$

For simplicity, either (hk0) can be used to give a or (00l) can be used to give c. The signal splitting is pronounced for the (110) reflection in the region of 29–33° and this reflection was used to calculate the La^{3+}:Eu^{3+} ratio. The contribution of both elements to each peak was determined via Vegard's law since both diffractions are the same crystal structure. According to Vegard's law, the lattice parameters of a solid solution is approximately the mean of the two lattice parameters, expressed by Eq. S8.[1–3]

$$a_{La\,+\,Eu} = (1 - x) * a_{La} + x * a_{Eu}\ (Eq.\,S8)$$

Where a_{La+Ln} is the average lattice parameter a of the alloy, a_{La} the La lattice parameter and a_{Eu} the Eu lattice parameter. The elemental fraction is expressed by x.

2. Characterization of Spent Catalyst Materials

During the methane oxychlorination (MOC) reaction, catalyst chlorination occurs and a bulk phase transition from LnOCl to $LnCl_3$ can take place (or at least partly). A dechlorination step in 2:4:1:15 CH_4:O_2:N_2:He was performed at 550 °C to induce a phase transition of the material from the chlorinated phase to LnOCl, thereby removing excess chlorine in the catalyst material and making the sample air-stable. Subsequently, post-characterization of the catalyst materials with N_2 physisorption, XRD and TEM is performed. However, the physicochemical properties obtained after the post-characterization of the active catalyst material might not be representative of the active catalyst material in the reaction. Nevertheless, the dechlorination step has practical considerations, and without, no post-characterization could be performed. Lanthanide chlorides are hygroscopic in nature and, when exposed to air, form their corresponding hydrates. Upon rehydration, the structure of the catalyst material can be lost as e.g. lanthanum chloride dissolves from moisture in the air. TEM measurements cannot be performed under inert conditions. Furthermore, for XRD and N_2 physisorption, it implies that the catalyst material has to be transported to an inert atmosphere to guarantee the preservation of the physicochemical properties of the active catalyst material. The reactor set-up does not allow us to close the reaction tube and prevent rehydration. Even though the reactor tube can be transferred to a glovebox, we cannot assure that rehydration did not occur. The potential rehydration raises an issue as sorption samples are typically dried at elevated temperatures under vacuum conditions. During this pretreatment, the thermal dehydration can cause hydrolysis of the lanthanide chloride to the lanthanide oxychloride and release HCl.[4,5] The sorption apparatus used in our laboratory is not corrosion resistant and thus the experiment would perform harm to the equipment. XRD can be performed under inert conditions, but its XRD pattern is difficult to analyze as there are many unidentifiable diffractions. Due to these practical considerations, we chose to perform the dechlorination step, as we believe that still some qualitative trends can be deducted from these results. However, we do acknowledge the fact that the physicochemical properties of the catalyst material could be altered during this dechlorination step.

3. Additional Experimental Data

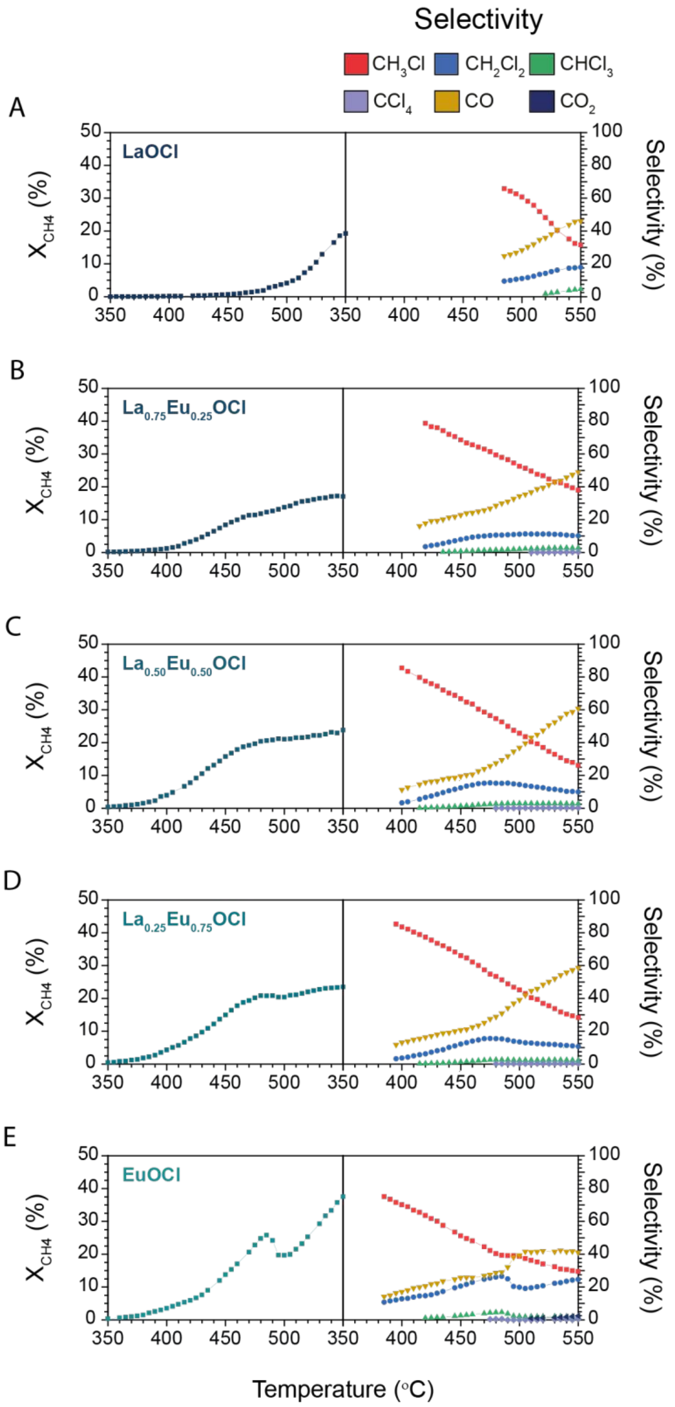

Figure S1. CH_4 conversion (X_{CH4}) and the corresponding selectivity plotted versus the reaction temperature for (A) LaOCl, (B) $La_{0.75}Eu_{0.25}OCl$, (C) $La_{0.50}Eu_{0.50}OCl$, (D) $La_{0.25}Eu_{0.75}OCl$ and (E) EuOCl. La^{3+}-Eu^{3+} solid solution catalysts show similar trends in their catalytic performance, which is different compared to their monometallic counterparts. Reaction conditions: CH_4:HCl:O_2:N_2:He of 2:2:1:1:14 (in mL/min) from 350-550 °C with a ramp rate of 1 °C/min. Selectivity is given when CH_3Cl and CO were above the detection limit of the GC.

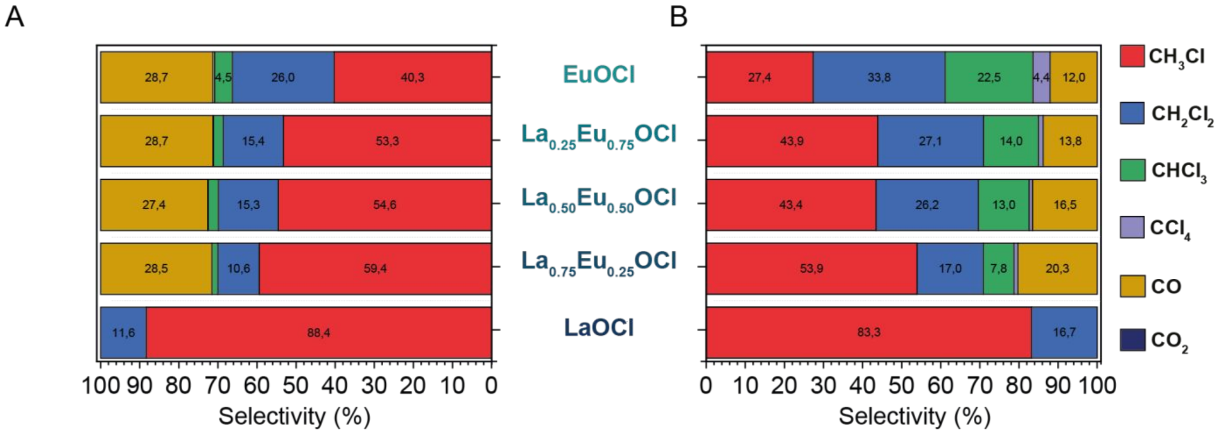

Figure S2. Selectivity towards CH_3Cl (red), CH_2Cl_2 (blue), $CHCl_3$ (green), CCl_4 (purple), CO (yellow), and CO_2 (dark blue) for LaOCl, $La_{0.75}Eu_{0.25}OCl$, $La_{0.50}Eu_{0.50}OCl$, $La_{0.25}Eu_{0.75}OCl$ and EuOCl tested under (A) 10% HCl and (B) 80% HCl in the feed. La^{3+}-Eu^{3+} solid solution catalysts show very similar selectivity under the same conditions for both the 10% and 80% HCl in the feed. The observed selectivity in both cases varies drastically compared to the selectivity observed for LaOCl and EuOCl. Reaction conditions: CH_4:HCl:O_2:N_2:He of 2:2:1:1:14 (10% HCl, in mL/min) or 2:16:1:1:0 (80% HCl, in mL/min), 480 °C.

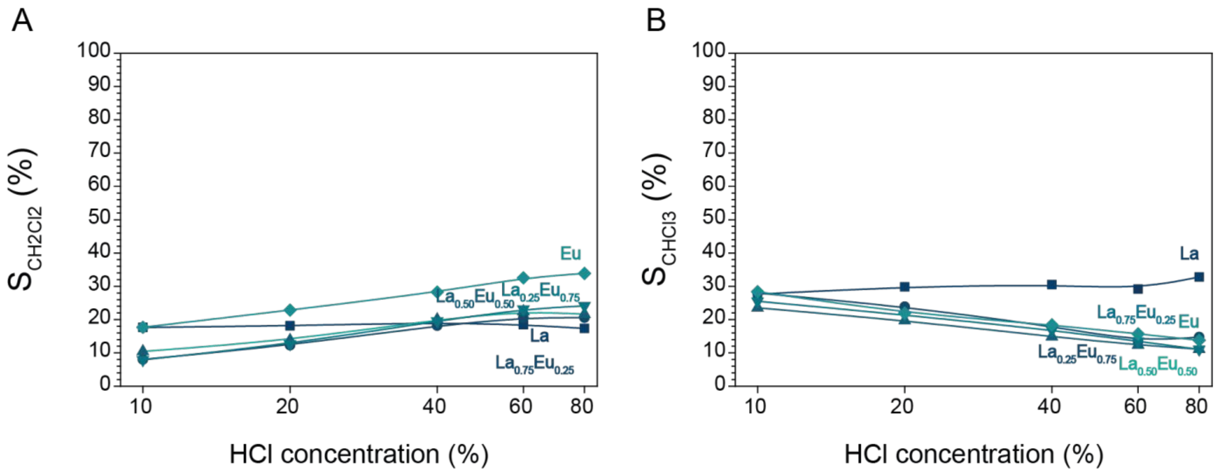

Figure S3. Selectivity towards (A) CH_2Cl_2 (S_{CH2Cl2}) and (B) $CHCl_3$ (S_{CHCl3}) versus the HCl concentration for LaOCl (T = 520 °C), $La_{0.75}Eu_{0.25}OCl$ (T = 475 °C), $La_{0.50}Eu_{0.50}OCl$ (T = 450 °C), $La_{0.25}Eu_{0.75}OCl$ (T = 450 °C) and EuOCl (T = 450 °C) in the methane oxychlorination (MOC) reaction. The three La^{3+}-Eu^{3+} solid solution catalysts show similar S_{CH2Cl2} as well as S_{CO}. The temperature was adjusted to reach X_{CH4} = 10% for CH_4:HCl:O_2:N_2:He of 2:2:1:1:14. When stable conversion was reached, the HCl:He ratio was adjusted so that the HCl concentration was increased to 20%, 40%, 60% and 80%, while keeping a constant flow of 20 mL/min.

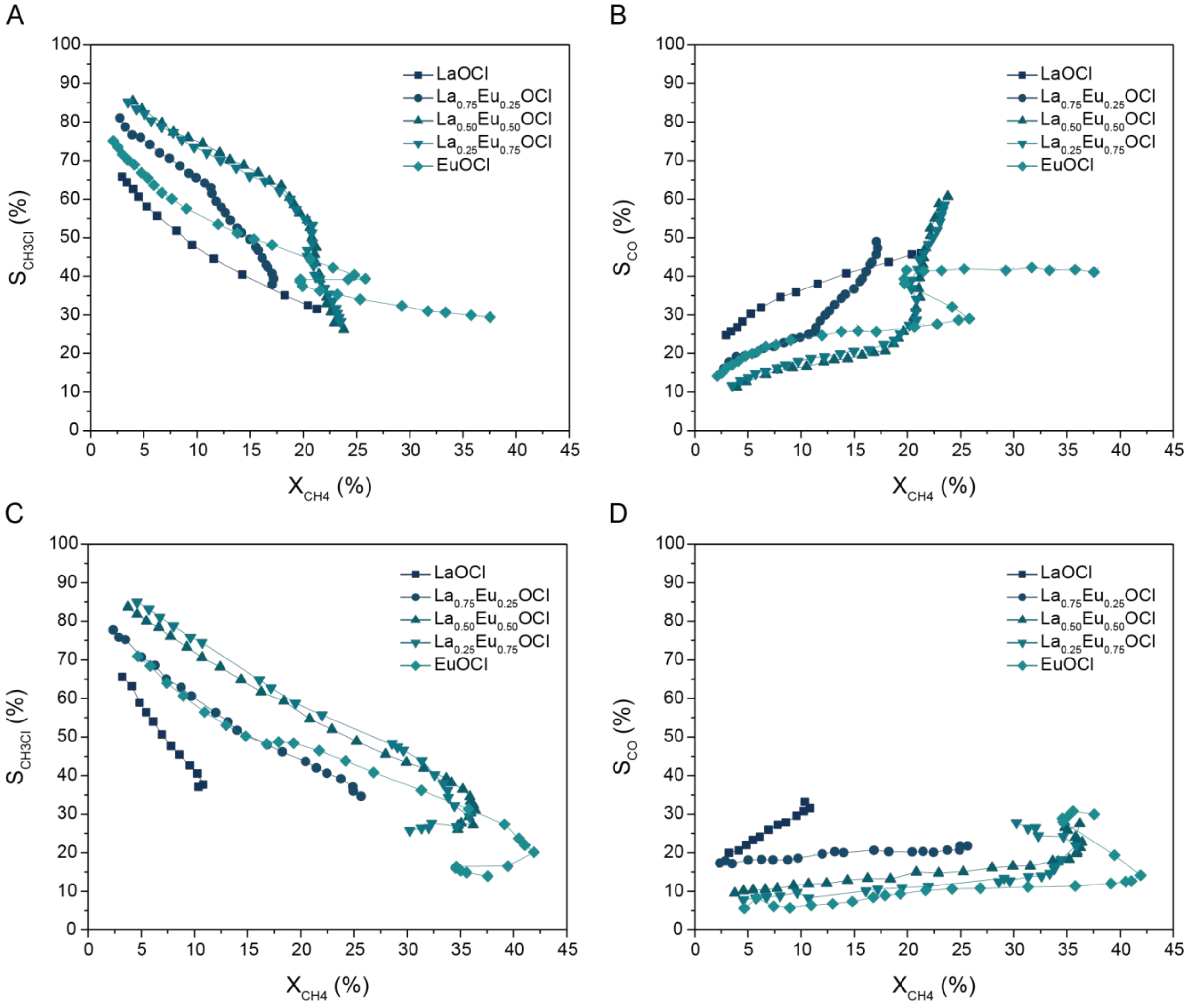

Figure S4. Non-isothermal conversion-selectivity plots for LaOCl, $La_{0.75}Eu_{0.25}OCl$, $La_{0.50}Eu_{0.50}OCl$, $La_{0.25}Eu_{0.75}OCl$ and EuOCl. The conversion (X_{CH4}) - CH_3Cl selectivity (S_{CH3Cl}) is given for (A) 10% HCl in the feed and (C) 80% HCl in the feed. The corresponding X_{CH4}-CO selectivity (S_{CO}) is also given for (B) 10% HCl in the feed and (D) 80% HCl in the feed. Reaction conditions: CH_4:HCl:O_2:N_2:He of 2:2:1:1:14 (10% HCl, in mL/min) or 2:16:1:1:0 (80% HCl, in mL/min), temperature ranging from 350 – 550 °C.

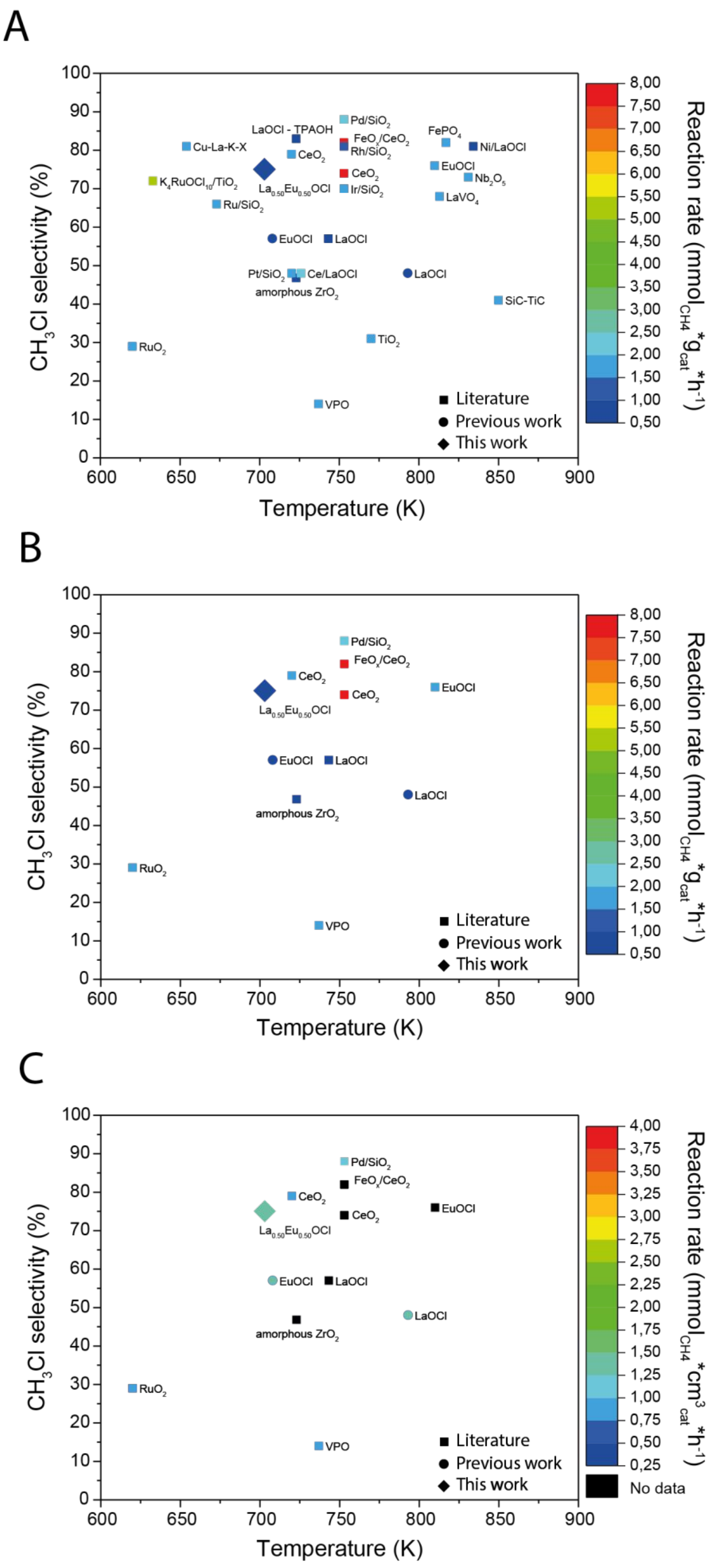

Figure S5. (A) The selectivity plotted versus the temperature at which 10% CH_4 conversion is reached for the catalyst systems of Table S1. The color of the symbol represents the reaction rate, normalized to the catalyst weight ($g_{catalyst}$). (B) The selectivity plotted versus the temperature at which 10% CH_4 conversion is reached for the catalytic systems of Table S1 where stable (chemical, structural and/or catalytic) performance is reported. (C) Same plot as (B) but normalized to the bed volume (in $cm^3_{catalyst}$) instead of catalyst weight.

Table S1. Catalytic systems reported in the academic literature. Temperature, CH_3Cl selectivity, CO selectivity and reaction rate are given at 10% CH_4 conversion. Subsequently, the reported stability (chemical, structural and/or catalytic) is tabulated. Only the catalytic systems that were reported as exhibiting no stability issues were graphically depicted in Figure S5B.

Catalytic System	Temperature (K)	CH_3Cl selectivity (%)	CO selectivity (%)	Reaction rate ($mmol_{CH4}*g_{cat}*h^{-1}$)	Reaction rate ($mmol_{CH4}*cm^3_{bed}*h^{-1}$)	Remark on stability	Ref
LaOCl	743	57	35	0,99	-	Stable	[6,7]
LaOCl - TPAOH	723	83	8,4	0,99	-	Not reported	[8]
Ni/LaOCl	834	81	12	0,99	-	Dopant loss	[7]
Ce/LaOCl	723	48	48	0.99	-	Dopant loss	[7]
CeO_2	753	74	-	7,88	-	Stable	[9]
FeO_x-CeO_2	753	82	-	7,88	-	Stable	[9]
K_4RuOCl_{10}/ TiO_2	633	72	13	5.11	-	Not reported	[10]
RuO_2	620	29	67	1,59	0,88	Known stablility [11]	[12]
CeO_2	720	79	11	1,59	0,88	Known stability [9]	[12]
$LaVO_4$	813	68	31	1,59	0,88	Not reported	[12]
Nb_2O_5	831	73	26	1,59	0,88	Not reported	[12]
TiO_2	770	31	68	1,59	0,88	Not reported	[12]
VPO	737	14	84	1,59	0,88	Stable	[12]
EuOCl	810	76	12	1,59	0,88	Known stablility [13]	[14]
Cu-La-K-X	654	81	9	1,59	0,88	Not stable	[15]
$FePO_4$	817	82	18	1,59	0,88	Not reported	[15]
SiC-TiC	850	41	55	1,59	0,88	Not reported	[16]
amorphou s ZrO_2	723	48	32	2,46	-	Stable	[17]
Ru/SiO_2	673	66	27	1,98	0,99	$RuSi_x$ formation	[18]
Pt/SiO_2	723	48	48	1,59	0,79	PtO_2 formation	[18]
Ir/SiO_2	753	70	20	1,74	0,87	Ir and IrSi formation	[18]
Rh/SiO_2	753	81	11	1,27	0,63	Rh formation	[18]
Pd/SiO_2	753	88	6	2,38	1,19	Stable	[18]
LaOCl	793	48	36	0.99	1,46	Stable [6,7]	[13]
EuOCl	708	57	23	0.99	1,46	Stable	[13]
LaEuOCl	703	75	16	0.99	1,46	Stable	-

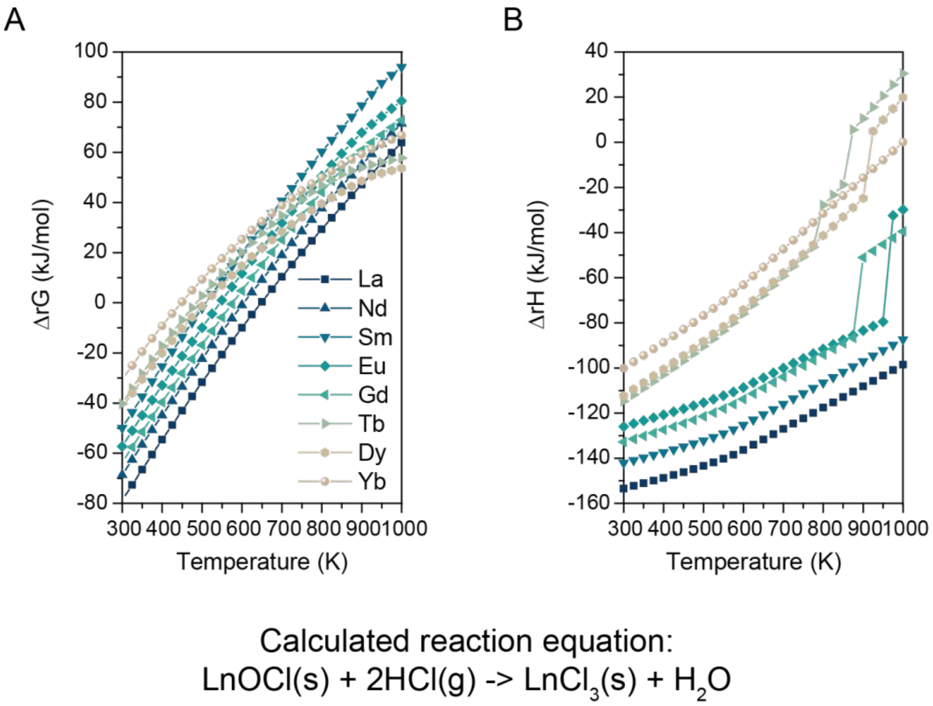

Figure S6. Calculated (A) Gibbs free energy ($\Delta_r G$) and (B) enthalpy ($\Delta_r H$) of the chlorination of LnOCl to $LnCl_3$. Of all the lanthanides available in HSC Chemistry for the chlorination reaction given above, La^{3+} was found to have the lowest $\Delta_r G$ and $\Delta_r H$. This indicated that the chlorination of LaOCl to $LaCl_3$ is the most facile compared to the other lanthanides. Thermodynamic calculations were performed with HSC chemistry 7.1. The reaction equation given in the figure was filled in for every lanthanide element as input in the Chemical Reactions Calculator and the $\Delta_r G$ and $\Delta_r H$ between 300K and 1000K with steps of 25K were calculated.

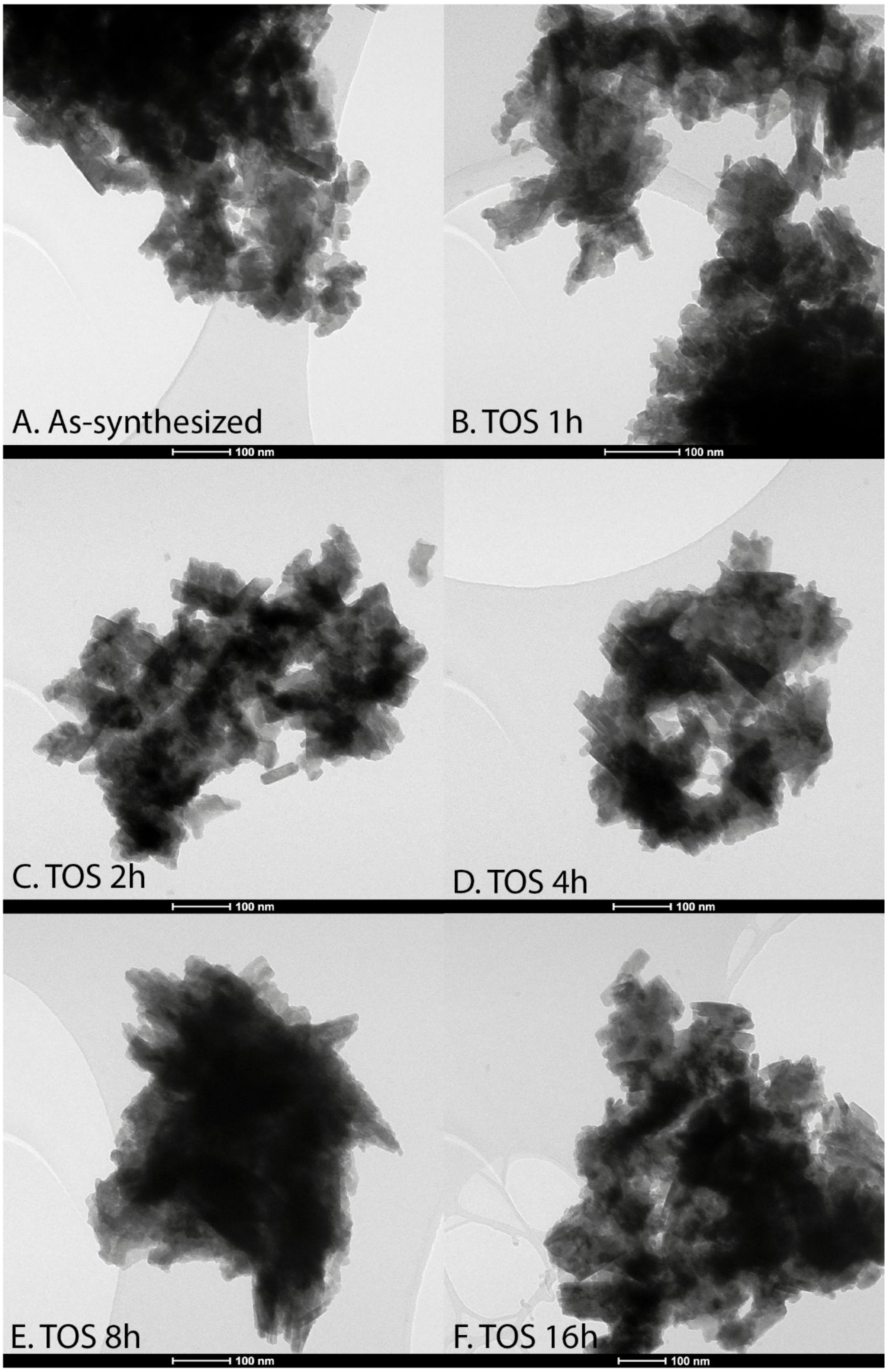

Figure S7. Bright field Transmission Electron Microscopy (TEM) performed on the $La_{0.50}Eu_{0.50}OCl$ catalyst materials used for the time series in Figure 5. Fresh catalyst was loaded into the reactor for every measurement and the TEM images are from (A) as-synthesized, (B) 1h, (C) 2, (D) 4, (E) 8 and (F) 16h time on stream (TOS) $La_{0.50}Eu_{0.50}OCl$ at 450 °C. Dechlorination step was performed to remove hygroscopic $LnCl_3$.

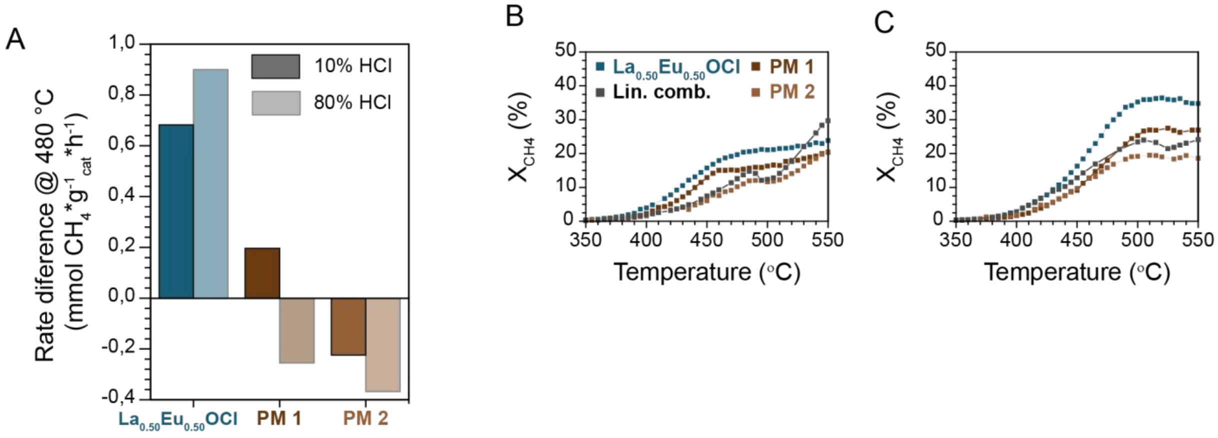

Figure S8. (A) The rate difference of $La_{0.50}Eu_{0.50}OCl$, PM1 and PM2 with respect to the linear combination of LaOCl and EuOCl. The CH_4 conversion rate (X_{CH4}) of $La_{0.50}Eu_{0.50}OCl$, PM1 and PM2 and the linear combination of LaOCl and EuOCl are plotted versus temperature under (B) 10% HCl and (C) 80% HCl. Physical mixture 1 (PM1) was prepared by sonication of LaOCl and EuOCl nano powders in ethanol, after which the solvent was evaporated at 80 °C in static air and the powder was sieved (125-425 μm size fraction). Intimate mixing of the powders is achieved, but no solid solution is made. Physical mixture 2 (PM2) was prepared by mixing sieved LaOCl and EuOCl particles (125-425 μm size fraction), hence no intimate contact is expected. Only when intimate contact between La^{3+} and Eu^{3+} is present, enhancement of activity is observed.

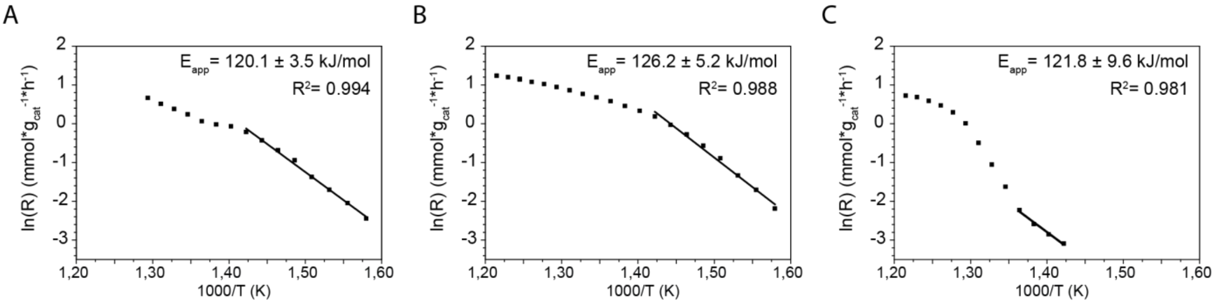

Figure S9. Apparent activation energy (E_{app}) of the methane oxychlorination (MOC) reaction over (A) EuOCl, (B) $La_{0.50}Eu_{0.50}OCl$ and (C) LaOCl using 10% HCl in the feed. Here, 250 mg of catalyst material (125–425 µm sieve fraction) was used to determine the apparent activation energy below 10% conversion to adhere to the requirements of the differential reaction model. Reaction conditions: CH_4:HCl:O_2:N_2:He of 2:2:1:1:14 (10% HCl, in mL/min), temperature ramped from 350-550 °C with steps of 10 °C and kept at every step for 45 minutes.

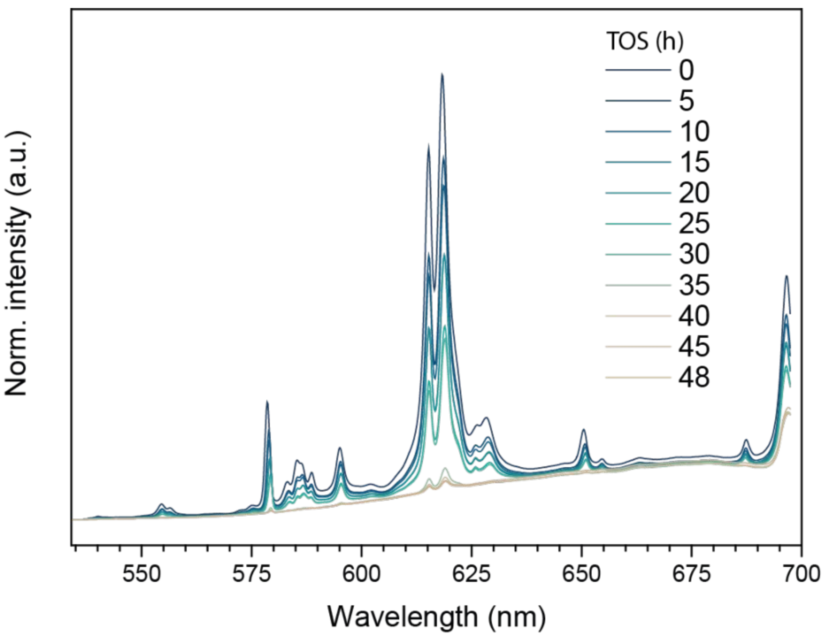

Figure S10. Photoluminescence spectra of $La_{0.50}Eu_{0.50}OCl$ of times corresponding to the runtimes in Figure 7. A gradual decrease in spectral intensity was observed, indicating that EuOCl was chlorinated to $EuCl_3$. Still, after 48 h, features corresponding to the luminescence signal of EuOCl were distinguishable, indicating that the catalyst was not entirely chlorinated. For reaction conditions, see Figure 7.

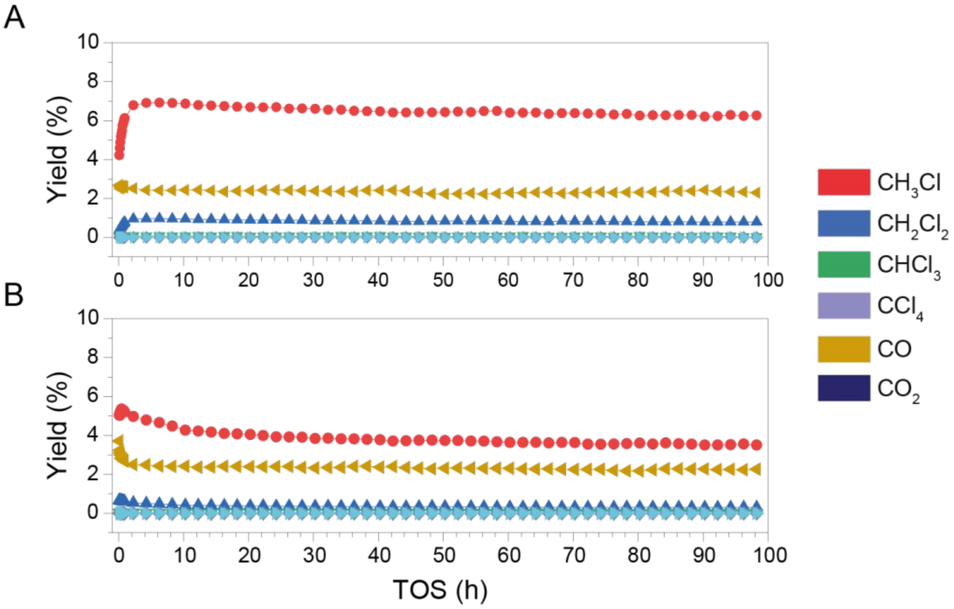

Figure S11. Catalytic stability of (A) $La_{0.50}Eu_{0.50}OCl$ and (B) EuOCl in the methane oxychlorination reaction tested for 100h time on stream (TOS) at 450 °C. Both catalyst materials show stable performance during the duration of the experiment. Reaction conditions: CH_4:HCl:O_2:N_2:He of 2:2:1:1:14 (10% HCl, in mL/min), temperature 450 °C.

References

(1) King, H. W. Quantitative Size-Factors for Metallic Solid Solutions. *J. Mater. Sci.* **1966**, *1*, 79–90. https://doi.org/10.1007/BF00549722.

(2) Vegard, L. Die Konstitution Der Mischkristalle Und Die Raumfullung Der Atome. *Z. fur Phys.* **1921**, *5*, 17–26. https://doi.org/10.1007/BF01349680.

(3) Zhong, X.; Feng, Y.; Knoll, W.; Han, M. Alloyed $Zn_xCd_{1-x}S$ Nanocrystals with Highly Narrow Luminescence Spectral Width. *J. Am. Chem. Soc.* **2003**, *125*, 13559–13563. https://doi.org/10.1021/ja036683a.

(4) Lyle, S. J.; Westall, W. A. A Study of the Thermal Decomposition of Hydrated Europium(III) Chloride and Europrium(III) Bromide. *Thermochim. Acta* **1983**, *68*, 51–58. https://doi.org/10.1016/0040-6031(83)80379-7.

(5) Roy, R. J.; Kipouros, G. J. Estimation of Vapour Pressures of Neodymium Trichloride Hydrates. *Thermochim. Acta* **1991**, *178*, 169–183. https://doi.org/10.1016/0040-6031(91)80308-6.

(6) Peringer, E.; Podkolzin, S. G.; Jones, M. E.; Olindo, R.; Lercher, J. A. $LaCl_3$-Based Catalysts for Oxidative Chlorination of CH_4. *Top. Catal.* **2006**, *38*, 211–220. https://doi.org/10.1007/s11244-006-0085-7.

(7) Peringer, E.; Salzinger, M.; Hutt, M.; Lemonidou, A. A.; Lercher, J. A. Modified Lanthanum Catalysts for Oxidative Chlorination of Methane. *Top. Catal.* **2009**, *52*, 1220–1231. https://doi.org/10.1007/s11244-009-9265-6.

(8) Peringer, E.; Tejuja, C.; Salzinger, M.; Lemonidou, A. A.; Lercher, J. A. On the Synthesis of $LaCl_3$ Catalysts for Oxidative Chlorination of Methane. *Appl. Catal. A Gen.* **2008**, *350*, 178–185. https://doi.org/10.1016/j.apcata.2008.08.009.

(9) He, J.; Xu, T.; Wang, Z.; Zhang, Q.; Deng, W.; Wang, Y. Transformation of Methane to Propylene: A Two-Step Reaction Route Catalyzed by Modified CeO_2 Nanocrystals and Zeolites. *Angew. Chem. Int. Ed.* **2012**, *51*, 2438–2442. https://doi.org/10.1002/anie.201104071.

(10) Shalygin, A.; Paukshtis, E.; Kovalyov, E.; Bal'zhinimaev, B. Light Olefins Synthesis from C1-C2 Paraffins via Oxychlorination Processes. *Front. Chem. Sci. Eng.* **2013**, *7*, 279–288. https://doi.org/10.1007/s11705-013-1338-1.

(11) Over, H. Atomic-Scale Understanding of the HCl Oxidation over RuO_2, a Novel Deacon Process. *J. Phys. Chem. C* **2012**, *116* (12), 6779–6792. https://doi.org/10.1021/jp212108b.

(12) Paunović, V.; Zichittella, G.; Verel, R.; Amrute, A. P.; Pérez-Ramírez, J. Selective Production of Carbon Monoxide via Methane Oxychlorination over Vanadyl Pyrophosphate. *Angew. Chem. Int. Ed.* **2016**, *55*, 15619–15623. https://doi.org/10.1002/anie.201608165.

(13) Terlingen, B.; Oord, R.; Ahr, M.; Hutter, E.; van Lare, C.; Weckhuysen, B. M. Mechanistic Insights into the Lanthanide-Catalyzed Oxychlorination of Methane as Revealed by Operando Spectroscopy. *ACS Catal.* **2021**, *11*, 10574–10588. https://doi.org/10.1021/acscatal.1c00393.

(14) Zichittella, G.; Aellen, N.; Paunović, V.; Amrute, A. P.; Pérez-Ramírez, J. Olefins from Natural Gas by Oxychlorination. *Angew. Chem. Int. Ed.* **2017**, *56*, 13670–13674. https://doi.org/10.1002/anie.201706624.

(15) Zichittella, G.; Paunović, V.; Amrute, A. P.; Pérez-Ramírez, J. Catalytic Oxychlorination versus Oxybromination for Methane Functionalization. *ACS Catal.* **2017**, *7*, 1805–1817. https://doi.org/10.1021/acscatal.6b03600.

(16) Zichittella, G.; Puértolas, B.; Siol, S.; Paunović, V.; Mitchell, S.; Pérez-Ramírez, J. An Activated TiC-SiC Composite for Natural Gas Upgrading via Catalytic Oxyhalogenation. *ChemCatChem* **2018**, *10*, 1282–1290. https://doi.org/10.1002/cctc.201701632.

(17) Huang, J.; Wang, W.; Li, D.; Xu, S.; Liu, Q.; Chen, X.; Fei, Z.; Zhang, Z.; Cui, M.; Tang, J.; Qiao, X. Facile Construction of Non-Crystalline ZrO_2 as an Active yet Durable Catalyst for Methane Oxychlorination. *J. Sol-Gel Sci. Technol.* **2019**, *92*, 163–172. https://doi.org/10.1007/s10971-019-05089-x.

(18) Paunović, V.; Zichittella, G.; Hemberger, P.; Bodi, A.; Pérez-Ramírez, J. Selective Methane Functionalization via Oxyhalogenation over Supported Noble Metal Nanoparticles. *ACS Catal.* **2019**, *9*, 1710–1725. https://doi.org/10.1021/acscatal.8b04375.
